# Development, Testing, and Thermoforming of Thermoplastics Reinforced with Surface-Modified Aramid Fibers for Cover of Electronic Parts in Small Unmanned Aerial Vehicles Using 3D-Printed Molds

**DOI:** 10.3390/polym16152136

**Published:** 2024-07-27

**Authors:** Maria Sonmez, Cristina-Elisabeta Pelin, George Pelin, Bogdan Rusu, Adriana Stefan, Maria Daniela Stelescu, Madalina Ignat, Dana Gurau, Mihai Georgescu, Mihaela Nituica, Ovidiu-Cristian Oprea, Ludmila Motelica, Bartłomiej Waśniewski, Paweł Ortyl, Roxana Doina Trușcă

**Affiliations:** 1 INCDTP-ICPI—National Research and Development Institute for Textile and Leather—Division Leather and Footwear Research Institute, Ion Minulescu St. 93, 031215 Bucharest, Romania; ficaimaria@yahoo.com (M.S.); dmstelescu@yahoo.com (M.D.S.); madalina.fleancu@yahoo.com (M.I.); danagurau84@gmail.com (D.G.); mihai.georgesku@yahoo.com (M.G.); mihaelavilsan@yahoo.com (M.N.); 2 INCAS—National Institute for Aerospace Research “Elie Carafoli”, Bd. Iuliu Maniu 220, 061126 Bucharest, Romania; pelin.george@incas.ro (G.P.); rusu.bogdan@incas.ro (B.R.); stefan.adriana@incas.ro (A.S.); 3 Faculty of Chemical Engineering and Biotechnologies, National University of Science and Technology Politehnica Bucharest, 1-7 Polizu St., 011061 Bucharest, Romania; ovidiu73@yahoo.com (O.-C.O.); motelica_ludmila@yahoo.com (L.M.); 4 Academy of Romanian Scientists, 3 Ilfov St., 050044 Bucharest, Romania; 5 Centre for Composite Technologies, Lukasiewicz Research Network—Institute of Aviation, Krakowska 110/114, 02-256 Warsaw, Poland; bartlomiej.wasniewski@ilot.lukasiewicz.gov.pl (B.W.); pawel.ortyl@ilot.lukasiewicz.gov.pl (P.O.); 6 National Centre for Micro and Nanomaterials and National Centre for Food Safety, National University of Science and Technology Politehnica Bucharest, 060042 Bucharest, Romania; truscaroxana@yahoo.com

**Keywords:** fiber surface modification, aramid fibers, thermoplastic polymers, mechanical properties, vacuum thermoforming, 3D-printed molds, unmanned aerial vehicle applications

## Abstract

This paper presents the development, characterization, and testing of PP/PE-g-MA composites with 10 and 15 wt% surface-modified aramid fibers, and aluminum-based pigment, as covers for a small drone body for collision protection. The successful fiber surface modification with SiO_2_ by the sol–gel method using TEOS was confirmed by FTIR, SEM, and EDS analyses. The composites were characterized by FTIR and SEM analyses and surface energy and water contact angle measurements and tested in terms of tensile, flexural, impact, and thermal properties. The materials exhibited hydrophobic character and compact and uniform morphostructures, with increased surface energy with fiber content owed to improved adhesion between modified fibers and the matrix. Compared to the control sample, composites with modified fibers showed an increase by 20% in tensile strength, and 36–52% in the modulus, and an increase by 26–33% in flexural strength and 30–47% in the modulus, with higher values at room temperature. Impact resistance of modified fiber composites showed an increase by 20–40% compared to the control sample, due to improved interaction between SiO_2_-modified fibers and maleic anhydride, which inhibits crack formation, allowing higher energies’ absorption. The composites were vacuum-thermoformed on 3D-printed molds as a two-part cover for the body of a drone, successfully withstanding the flight test.

## 1. Introduction

Nowadays, the main techniques for manufacturing finished parts made of polymer-based materials (stamp forming, vacuum forming, vacuum-assisted resin infusion/transfer molding, etc.) [1,2] have a common central major element, represented by the mold, which is the most important tooling as it supports the development of products with the required shape, size, and intricate details [3,4,5]. Traditional tooling is produced through metallic molds (such as aluminum or steel alloys), developed by conventional subtractive fabrication methods, in which the melted metal is poured into a block having the rough geometry and size of the desired product, and intricate details are adjusted using drills and polishers with computerized numeric control (CNC). Due to high durability, metallic molds are one of the most appropriate for the large-scale production of up to 100,000 parts. However, since the associated time and costs are high (reaching amounts of USD 10,000 over several months’ lead times) [6], they do not represent a solution for smaller production volumes. Other materials used to develop molds by subtractive techniques, especially for vacuum forming, are wood and structural foams, but the level of intricate details and rapid deformation can generate problems.

Given that the mold is the key factor in the vacuum forming processes, as it dictates the final design of the product, the materials and technologies used to produce it exhibit a tremendous influence affecting the precision, accuracy, complexity, and total time for the production of a final part. New digitalized unconventional methods to develop tooling and molds such as additive manufacturing, commonly known as 3D printing, are becoming more attractive, especially for areas in which traditional methods exhibit drawbacks [7]. Using additive-manufactured tooling contributes to the supply chain and productivity optimization, as it enables the rapid and high-complexity customization of products, with low-cost investments and reduced lead times, creating complementary solutions for the manufacturing environment where the requirements cannot be fulfilled by traditional tooling.

Using polymers as the material for producing 3D-printed tooling brings significant advantages in some technological and business cases such as low-volume production, especially when the parts produced exhibit high-complexity geometries, requirements of design flexibility due to several reiteration steps for product upgrading or functionality improvement, and requirements related to the urgent production of parts and associated tooling, in single-case use of customizable products (such as patient-customized prosthetics) [8]. Besides this, most materials suitable for milling are difficult to be re-machined into different geometries; once the tool reaches its end-of-life it becomes waste, as opposed to the tooling manufactured by 3D printing that generally belongs to the recyclable thermoplastic class.

Thermoplastic polymers are often processed through melt extrusion and injection, representing two of the oldest and most used technologies, extremely suitable for “bulk” products, but less adequate for thin products, such as sheets or foils. Vacuum forming or thermoforming is a conventional processing technique of thin thermoplastic foils, which involves pressing heated plastic foils or films over a mold using atmospheric pressure [9], resulting in tridimensional parts that are reversed replicas of the mold geometry. Thermoforming can be performed by three main routes: 1. Vacuum—for parts that are precisely formed on one side; 2. Pressure (approximately 6.9 bar)—for parts with complex and complicated details, which require surface finish quality similar to injection-molded parts; 3. Mechanical forming—for deep profiles, by pressing negative/positive molds [10].

Overall, the most important stages of the thermoforming method are as follows: 1. The heating of the foil (to a temperature between the glass transition and the melting temperature, commonly referred to as softening temperature [11]), 2. The part formation using vacuum, compressed air, or an over-die, 3. The cooling (solidification) of the foil, 4. The extraction or demolding of the final part. Generally, thermoforming techniques are used in commercial and technical packaging industries, vacuum thermoforming being the main technology in wrapping products and encasement processing of products from consumer goods to connected devices and printed circuit boards [9]. The most commonly used thermoplastics in the packaging sector are polyethylene (PE) and polypropylene (PP) [12]. In the electronics sector, conventional and conductive polylactic acids were reported to be used as printed foils with conductive traces [9].

Composite materials are popular candidates in structural components used in aerospace, automotive, and other industries due to their high stiffness-to-weight and strength-to-weight ratios. Given the sustainability-related advantages of thermoplastics (good recyclability, fast-forming cycle, excellent shelf life), this class of composites is becoming a better choice in some situations, tending to replace the currently leading solution represented by thermosetting composite materials. Thermoforming is a suitable choice for thermoplastic composite development because it is a simple, fast, high-deformation process that can be easily accessed, automated, and used to make intricately shaped components [11,13]. For example, Striewe et al. [14] thermoformed a polyamide 6/glass fabric preform into a three-dimensional hat profile at approximately 270 °C, glued afterward with a closing plate in a locking box for impact resistance. Behrens et al. [15] developed a fully automated forming process to build a fiberglass-reinforced thermoplastic composite at 260 °C in a battery tray for a plug-in hybrid vehicle. Maron et al. [16] fabricated a thermoplastic composite shaft with an integrated flange from a preform tube within a temperature range higher than the melting point [11].

When designing 3D-printed tooling for use in thermoforming applications, one should consider the principles and requirements of both technologies, since the product replicates the mold’s geometry. The materials and methods selected for mold manufacturing depend on the product requirements and application, considering the production volume, complexity of parts, timeframe for manufacturing, and design versatility. The 3D-printed molds can ensure the same features as traditional ones but additionally, they allow design reiteration and tailoring with increased freedom, reproduction of geometries with a high level of detail accuracy and precision, and dimensional accuracy. Besides these, they can withstand up to several hundreds of cycles, and their shorter lead time significantly accelerates the manufacturing process requiring reduced personnel involvement compared to traditional tooling [17]. These advantages support the pressure that the thermoforming field faces, related to reducing manufacturing time, costs, and waste quantity, keeping the same level of product quality. Junk et al. thermoformed polyvinyl chloride and polystyrene sheets over 3D-printed molds, reducing overall costs by approximately 15% compared to aluminum molds [18]. Serrano-Mira et al. [19] obtained promising results on polylactic acid molds produced by fused deposition modeling for thermoforming PVC sheets to obtain tactile graphics.

The past decade has seen considerable growth in unmanned aerial vehicles (UAVs), commonly known as drones, and the continuous expansion of their applications, from the low-tech field of hobbies and Do-It-Yourself (DIY) to the high-tech field of security and military industries. Given their high susceptibility to collisions with different obstacles (buildings, trees, high-voltage poles, birds, other vehicles, etc.), drones can often suffer damage on structural or functional levels, affecting propellers but often extending to the main body. Therefore, studies focus on ensuring the further protection of sensitive or expensive elements. Even though unmanned vehicles are equipped with different onboard sensors for situational awareness and autonomous decision-making at run-time [20] and onboard mechanisms to avoid collisions with obstacles [21,22], due to their constantly increasing autonomy and ability to travel far from the base stations or operators, there is also an increasing need to build vehicles from suitable materials that can keep the damage to the minimum, in case of eventual collision. Designing and manufacturing UAVs are strongly guided by considering a series of loadings that could generate excessive elastic deformations, fracture, buckling, creeping, fatigue and impact failure, and corrosion, together with lightweight considerations [23].

Both vacuum thermoforming and 3D printing techniques are highly applicable with thermoplastic composites, for the field of UAVs, and other aviation applications [24]. Several companies already develop UAVs using thermoplastic composites and the stakeholders’ number is under constant growth. Oribi produces composite blades and propellers of drones and UAVs in a high volume [25], Celanese manufactures long and continuous fiber-reinforced thermoplastics with adequate properties for drones [26], Syensqo develops products using bio-based raw materials and recycled content and several unique thermoplastic alternatives suitable for small delivery drones [27], and Sabic offers a wide range of customized thermoplastic products for specific parts in drone structures [28].

The research domain is also interested in studying the use of thermoforming together with additively manufactured tooling. Junk et al. [29] proved that binder jetting-manufactured molds can be used for thermoforming a small number of UAVs’ cowling (up to 10 pieces); the developed molds are provided with channels and spacers already integrated in the CAD model, the total production time taking only an average of 20 h, with costs reduced by 23%. ABS sheets were successfully thermoformed, fixed, and cut to the cowling geometries. Other studies focused on the thermoforming behavior of high-density polyethylene reinforced with 0%, 20%, 30%, 40%, and 50% (*w*/*w*) sawdust particles to develop small blades of UAVs [30]. Erchiqui et al. [31] investigated the use of PET–hemp fiber composites for thermoforming applications, observing that the final thickness of the thermoformed part is not influenced by the constitutive equations of the investigated formulations, and fiber loading does not affect the time and energy required for the process. The same group investigated thermoforming of PMSQ-HDPE to manufacture a small-dimension NACA profile (a set of standardized airfoil shapes developed by National Advisory Committee for Aeronautics, widely used in aircraft wings design), proving the potential use of composites for the manufacture of drone blades by thermoforming [32].

This paper presents a study on the development, characterization, and testing of thermoplastic composites reinforced with aramid fibers that were previously subjected to surface modifications for improved compatibility with the matrix, and their vacuum thermoforming over 3D-printed polymeric molds, for the obtaining of a thin cover for the body of a small-size commercial drone. This paper approaches three scientific innovations: the surface functionalization of aramid fibers using a tetraethyl orthosilicate precursor to form SiO_2_ particles, the development and comprehensive characterization and testing of composites based on the modified fibers embedded into polyolefin blends as a matrix and aluminum-based pigment, and the final application of the developed materials as protection covers for the body of a small commercial drone, which includes the molds’ design and production via 3D printing, and the vacuum thermoforming of the composites in the form of thin sheets over the developed molds. Following properties’ evaluation and flight tests, the materials exhibited adequate mechanical, thermal, and morphological features to be recommended as potential candidates for the protection of drone parts, and the versatility and freedom allowed by the use of 3D-printed tooling sustain the extension towards parts and drones with different geometries or even products from other fields, which require similar properties for the materials they are manufactured from.

## 2. Materials and Methods

### 2.1. Raw Materials

All raw materials, both for changing the surface of aramid fibers and for making composites, were used without additional purifications.

Linear low-density polyethylene grafted with maleic anhydride (PE-g-MA)-type ADMER™ NF468E, from Mitsui Chemicals, Düsseldorf, Germany, with the following characteristics was used in the experiments: density of 0.92 g/cm^3^, melting point—120 °C, hardness—51 Shore D, izod impact strength (J/m^2^)—no break. Mastersafe MP-10-20B is an aluminum-based pigment produced by Eckart, Hartenstein, Germany, compatible with several plastic materials (including polyolefins), appearance: in the form of granules, color: silver/grey, and aluminum concentration (% *w*/*w*): ≥70–<90 (actual concentration is withheld as a trade secret), and was offered as a sample during the experiments by Nordmann, Rassmann, Bucharest, Romania S.R.L. The product DEUREX E 11 K is a wax based on non-polar polyethylene, in the form of fine granules, used in the plastics industry, with the role of a dispersing agent, lubricant, and demolding agent, having the following technical characteristics: drop point in °C—110–120, viscosity—140 °C, mPas—≤80, density (wax) in g/cm^3^—0.94–0.96, and produced by Deurex AG, Elsteraue, Germany. Tipplen K 948 polypropylene (impact copolymer) is produced by MOL Petrochemicals Co. Ltd., Tiszaújváros, Hungary. Aramid fibers with the commercial name Twaron, produced by Teijin, Tokyo, Japan, with the following technical characteristics were used: lengths of 250 µm and diameters between 8 and 10 µm. Tetraethyl orthosilicate (98%), density: 0.933 g/mL at 20 °C (lit.) and molecular weight: 208.33 g/mol, was purchased from Sigma-Aldrich, Darmstadt, Germany. Ethyl alcohol (96%), molecular weight: 46.07 g/mol, and ammonia solution (25%), molecular weight: 17.03 g/mol and density: 0.902 g/cm^3^, were purchased from CHIMREACTIV SRL, Bucharest, Romania. Acetone (99%) with M—58.8 g/mol was purchased from Cristal R Chim SRL, Bucharest, Romania. Polyethylene glycol, 4000, for synthesis, form: solid, mp: 53–58 °C, and density: 1.2 g/cm^3^ at 20 °C, and trisodium citrate, MW: 258.07 g/mol, melting point: 150 °C, and density: 1.815 g/cm^3^ (20 °C), were purchased from Sigma-Aldrich, Darmstadt, Germany.

**Figure 1:**
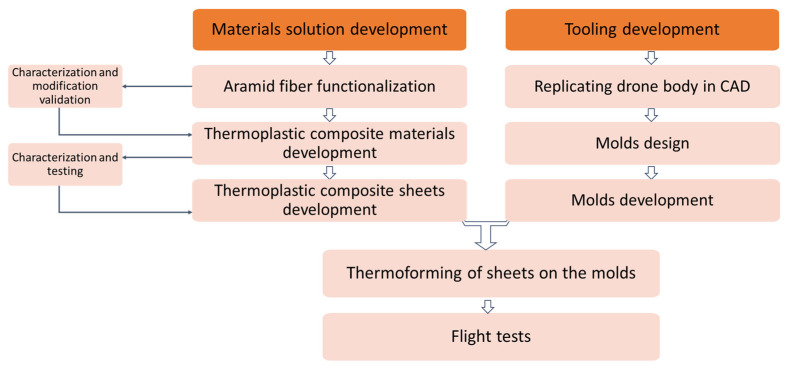
Overall organization of experimental stages within study.

Figure 1 illustrates a schematic representation of the development of the experimental study stages, for a more clear comprehension of the laboratory work.

### 2.2. Modification of Aramid Fiber Surface by Sol–Gel Technique

The surface of the aramid fibers was modified by the sol–gel method, using tetraethylorthosilicate as a precursor for the formation of SiO_2_ particles according to an adapted methodology published by Zang et al. [33] as follows: 15 g of aramid fibers washed with acetone and previously dried at 80 °C is placed in a plastic Berzelius glass for 24 h, over which 165 mL of TEOS (tetraethylorthosilicate, precursor for the synthesis of SiO_2_ particles) and 225 mL of ethyl alcohol (reaction medium) are added and ultrasonicated for 1 h (pulse mode, frequency of 80 kHz and power of 100%) at a temperature of 60 °C to cause the hydrolysis of TEOS with the formation of Si-OH species (gel is formed). In total, 210 mL of ethyl alcohol, 27 mL of distilled water, 15 mL of ammonia (having the role of condensing the Si-OH groups), 1.875 g of PEG (polyethylene glycol), and 1.875 g of trisodium citrate are added and sonicated for 5 h at 80 °C for the TEOS hydrolysis process and its condensation to take place, with the formation of SiO_2_ particles. In order for the transformation of all reactive SiOH species to take place by condensation, the mixture was kept at room temperature for 18 h, followed by washing with distilled water several times or until neutral pH, drying at 80 °C and 24 h, and pestling. Pestling of SiO_2_ particle-covered fibers is an important process in order to avoid their agglomeration in the matrix of PP/PE-g-MA. PEG and trisodium citrate were used to control the size of the SiO_2_ particles formed and to avoid their agglomeration. The amount of ammonia used in the synthesis has an important role in this hydrolysis/condensation process, because too low an amount would only lead to the partial hydrolysis of TEOS and to a reduced condensation capacity and implicitly to a low SiO_2_ yield. According to the synthesis method described above, 60 g of aramid fibers was modified and later used to make the composite materials presented in the following sections.

### 2.3. Composite Processing

Materials developed and investigated in this work, with the composition presented, were calculated so that the mixer chamber is filled to a proportion of 70% of its capacity (370 cm^3^). Mixtures were processed at 180 °C (temperature set on all Brabender areas), for 3 min at 80 rpm and 2 min at 130 rpm, to ensure the good dispersion of aramid fibers in the polymer matrix. After mixing was complete, the processed composites were taken out of the Brabender chamber and used to make plates to determine physical–mechanical properties and to make sheets/plates for thermoforming.

The materials developed and investigated in this study having the compositions presented in Table 1 were processed using a Brabender mixer, using the melt blending technique. In the case of mixtures containing unmodified/modified aramid fibers, prior to Brabender processing, a premix containing PP K948, PE-g-MA (50/50 ratio), and wax based on polyethylene (added in proportion of 5% relative to the amount of aramid fibers) is made. This mixture is heated in a hot-air oven, at the temperature of 110 °C (or until the wax becomes fluid); then, aramid fibers are gradually added under continuous stirring, so that the wax that has adhered to the surface of polymer granules captures the aramid fibers and distributes them evenly on their surface. This will ensure a good dispersion of aramid fibers in the polymer mass, thus avoiding their agglomeration. After this process, the pigment is added and the resulting mixture is introduced in the mixing chamber of the Brabender mixer. All mixtures presented were calculated so that the mixer chamber is filled to a proportion of 70% of its capacity (370 cm^3^). Mixtures were processed at 180 °C (temperature set on all Brabender areas), for 3 min at 80 rpm and 2 min at 130 rpm, to ensure the good dispersion of aramid fibers in the polymer matrix. After mixing was complete, the processed composites were taken out of the Brabender chamber and used to make plates to determine physical–mechanical properties and to make sheets/plates for thermoforming.

All mixtures in Table 1 were calculated so that the mixer chamber is filled to a proportion of 70% of its capacity (370 cm^3^). Mixtures were processed at 180 °C (temperature set on all Brabender areas), for 3 min at 80 rpm and 2 min at 130 rpm, to ensure the good dispersion of aramid fibers in the polymer matrix. After mixing was complete, the processed composites were taken out of the Brabender chamber and used to make plates to determine physical–mechanical properties and to make sheets/plates for thermoforming.

### 2.4. Production of Plates for Physical–Mechanical Tests and Films for Thermoforming

**Figure 2:**
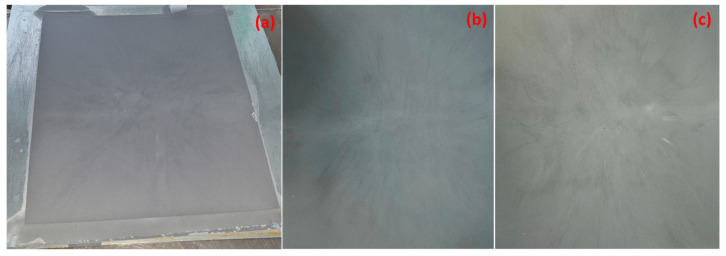
(**a**) Metallic mold (frame size of 340 × 300 mm and thickness of 0.5 mm) containing composite sheet after pressing. (**b**,**c**)—Sheet made from GFF 10% and GFF 15% mixtures after taking them out of mold and removing excess material.

Plates for determining physical–mechanical properties and sheets used for thermoforming the drone case were made using a Fontijne laboratory press, model: TP 600, with a maximum platen size of 400 mm × 400 mm, manufactured by Fontijne Grotnes, Vlaardingen, The Netherlands. The plates used for physical–mechanical determinations, with sizes of 15 cm × 15 cm and a thickness of 4 mm, were made according to the following parameters: platen temperature (upper and lower)—180 °C, pre-heating (no pressing)—3 min, pressing at 300 kN—2 min, and cooling—15 min. From the resulting plates, test specimens were punched using knives, according to standards in force (dumb-bell test specimens—ISO 527-1 [34]), to assess tensile behavior, and rod-shaped specimens with the sizes length—10 cm, thickness—4 mm, and width—8 mm to assess impact strength (ISO 179 A [35]) and flexural strength (ISO 178 [36]). Cylindrical specimens were used to determine the contact angle. FTIR microscopy, IR spectroscopy, DSC-TG analysis (were made on pieces left after punching specimens) and SEM microscopy, EDS, and optical microscopy were performed in the fracture cross-section of specimens after determining tensile strength. Sheets/films used in the thermoforming process were obtained in the electrical press at the same parameters as in the case of plates for physical–mechanical determinations. Figure 2a shows the metallic mold used to make the sheets and Figure 2b,c show sheets extracted from the mold processed from composites GFF 10% and GFF 15%. Sheets obtained had the following sizes: 340 mm × 300 mm.

### 2.5. Mold Design Development

Designing fabrication molds presents a significant challenge, especially when creating small, highly detailed, and precision parts. This complexity is evident when designing molds for a small commercial drone body, which will undergo a skin remake using a lighter thermoformed material with superior properties. The initial step in mold design involves accurately identifying the surface that needs to be reverse-engineered to obtain an identically shaped part. This process utilized CATIA V5 CAD software to model the drone body from a *.step file. Given the application of 3D printing technology, the mold geometry must be enclosed and meticulously designed to avoid the need for material supports, whose removal could compromise the aerodynamic surface.

**Figure 3:**
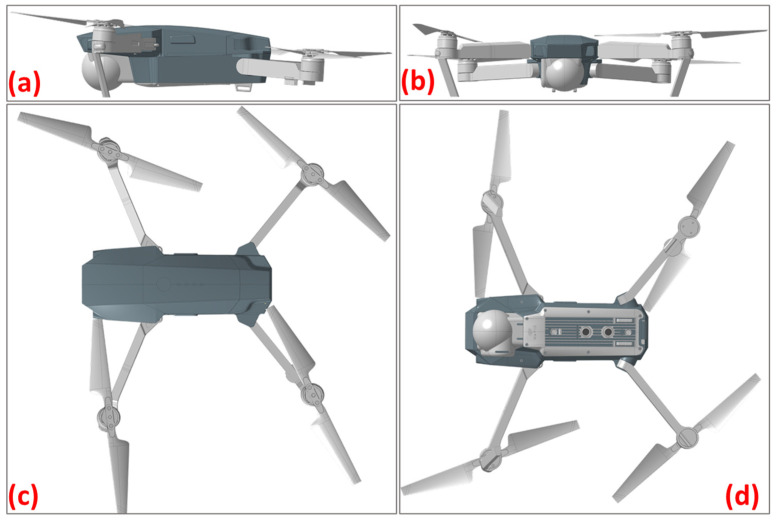
The drone’s body with the fitted cover visualized from different angles: (**a**) lateral view, (**b**) front view, (**c**) top view, (**d**) bottom view.

**Figure 4:**
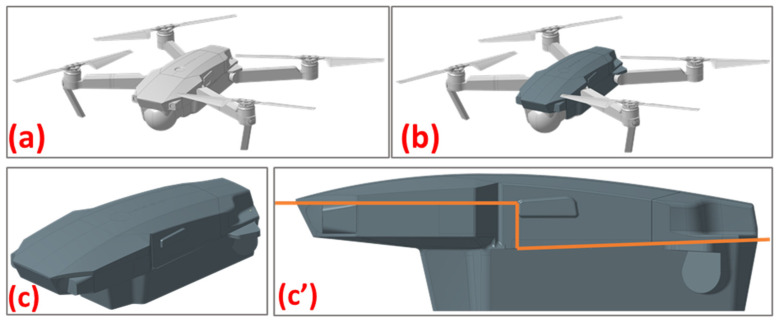
(**a**) The drone’s original body. (**b**) The drone’s body with the fitted cover. (**c**) The overall image of the cover. (**c’**) The section profile.

**Figure 5:**
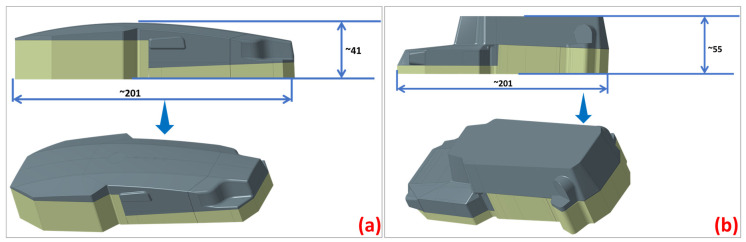
The section dividing (**a**) the upper mold and (**b**) the lower mold.

With the drone body model complete (Figure 3), the next step is to establish an engineering approach for the skin fabrication method (Figure 4). The thermoforming process imposes specific constraints that must be adhered to: avoiding sharp edges and cavities, using only positive molds with a limited height dictated by the press, and minimizing acute angles on the faces. Given the complex geometry of the model, the optimal solution was to create two separate molds: one for the lower body and one for the upper body (Figure 5). This approach minimizes the risk of defects such as cracking, warpage, and inconsistencies in dimensions or part thickness.

As part of an aerospace product with high expectations for life cycle sustainability, it is crucial to avoid thermoforming quality issues starting with the tooling design.

### 2.6. Molds’ Development

The tooling used for the thermoforming of the composite sheets was manufactured based on the chosen design presented above, the cover being composed of two molds with geometries corresponding to the upper and lower section of the drone. The molds were produced via additive manufacturing, through the fused filament fabrication (FFF) technique, widely known as fused deposition modeling (FDM), but the latter term is a Stratays Company registered trademark [37]. The 3D printer devices used were Markforged X7 Turbo, Watertown, MA, USA, an industrial printer that allows for the material extrusion process with a polymeric filament alone or with continuous fiber reinforcement. The material used for printing the molds was Onyx, a micro-carbon fiber-filled nylon, with a 71 MPa flexural strength, that is 1.4 times stronger and stiffer than ABS, setting the bar for surface finish, chemical resistivity, and heat tolerance (Table 2).

Onyx was chosen as a print material due to its versatility, as the advantages it already offers when printed alone (i.e., strength, toughness, chemical resistance) can be enhanced even more through the possibility to reinforce it with continuous fibers to yield aluminum-strength parts [38]. Table 3 presents the parameter used for the 3D printing of the two molds used to thermoform the two pieces that compose the cover of the drone’s body.

**Figure 6:**
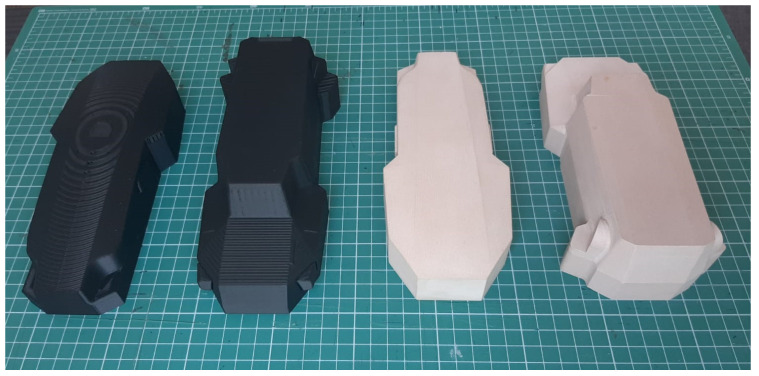
The molds used for the thermoforming of the drone cover: from left to right—3D-printed molds (**upper**,**lower**), CNC-machined molds (**upper**,**lower**).

It can be observed that the total printing time of the global mold used for the cover thermoforming was 28 h; this lead time can be varied, depending on the requirements of the molds to be developed. If rapid tooling development is needed, using higher printing speed values and lower infill density values can significantly reduce the total manufacturing time. If higher detail quality and precision are needed, lower printing speed values, different patterns, and higher numbers of layers and walls can lead to smoother surfaces and high detail accuracy of the printed part used as a mold. It is important to mention that the surface finish of the produced molds was not further polished (Figure 6left), as the resulting edges on the flat surface of the mold are an advantage for the thermoforming of the 0.5 mm thick composite sheets, imprinting further overall rigidity to the thin cover.

For comparison, traditional molds were also produced, through CNC machining of Obomodulan 700 PU, manufactured by OBO-Werke GmbH, Stadthagen, Germany a polyurethane foam, with 0.72 g/cm^3^, and a standard material used for model and master model development via CNC milling (Figure 6right). The smooth surface of these molds is an advantage when this feature is required for the thermoformed part on it; however, the complicated design in terms of CNC settings requires more steps along the process (including the change in mills’ type and diameters).

**Figure 7:**
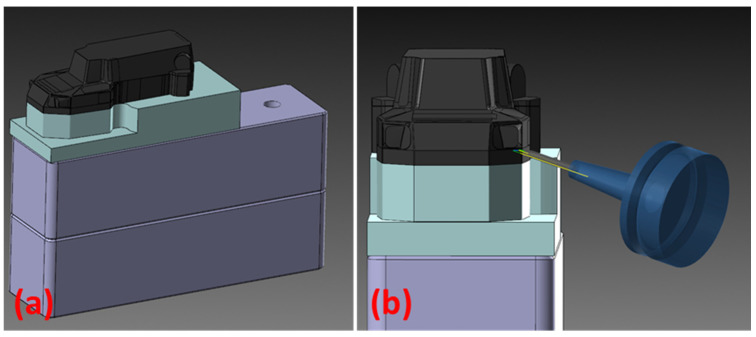
(**a**) A virtual representation of the lower mold design with the stock material mounted on the elevated platform. (**b**) A virtual representation of the machining process.

Two molds were made out of the Obomodulan 700 PU board, on a 5-axis Fanum Lambda GT milling machine, manufactured by Fanum Wielopole Skrzyńskie, Poland. Including the time required for setting up and preparing tool paths using CAM software, Mastercam 2022 version, the production time totals approximately 8 h per mold, the manufacturing process itself taking about 2 h of machining per mold. Stock material was mounted on an elevated platform (Figure 7), in order to enable machining some of the details, as they required the spindle axis to be set at angles above 90 degrees. The stock was fixed to the base using double-sided tape. The machining process used bull-nosed endmills for roughing, and ball endmills for finishing operations, starting from a 10 mm diameter down to a 0.5 mm diameter for the fine details.

### 2.7. Thermoforming of the Composite Sheets

**Figure 8:**
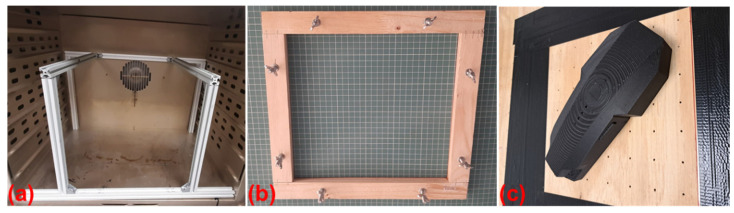
Elements that composed the custom-made thermoforming stand: (**a**) the metallic support with adjustable span length, which holds the wood frame with the sample inside the heating chamber, (**b**) the wooden frame that fits the composite sheet, (**c**) the upper 3D-printed mold positioned on the holed surface of the thermoforming table.

The composite sheets were vacuum-thermoformed on the developed molds, using a custom-made thermoforming stand (Figure 8) composed of a heating chamber (operating temperature of 5 ÷ 300 °C ± 2 °C) and a rectangular enclosing made of wood (table), which had the upper face provided with holes distributed over the entire surface and an air suction source connected on one of the lateral sides, providing a 0.8 bar vacuum pressure. The two pieces of the cover were made one at a time, the mold being placed over the surface with holes. The composite sheets were fixed with bolts on a wooden frame to be able to move them during the procedure. The sample pinned on the frame was inserted into the heating chamber at a temperature of 165 °C, visualizing the sheets’ appearance as the temperature rises. The average temperature at which the sheets began to deform plastically was 172 °C for the control samples and 173–178 °C for the aramid fiber-filled samples. At the moment the sheet deformed plastically, forming a uniform concavity, but without losing its integrity, the sample was transferred onto the thermoforming table with the simultaneous application of vacuum pressure through air suction. It is very important that the sheets do not reach the melting temperature value as it will not generate technical issues in manipulating the sheets and uniformly thermoform them. The sheets took the shape of the mold in 1–2 s.

### 2.8. Measurements

**Fourier transform infrared spectroscopy (FTIR)** was performed on equipment (NICOLET IS 50) from Thermo Scientific Company, Madison, WI, USA. All spectra were obtained in the ATR (Attenuated Total Reflectance) mode, at a resolution of 4 cm^−1^ and spectral domain of 4000–400 cm^−1^, conducting a number of scans, 32 scans/each sample. FTIR spectrometry was used to evaluate the surface of aramid fibers before and after modification with a SiO_2_ precursor, as well as to assess interactions occurring among the polymer matrix, aramid fibers, and other additives (wax based on PE, PE-g-MA, pigment) used to develop composites.

**The tensile and 3-point bending tests** were conducted on INSTRON 5982 equipment, Norwood, MA, USA provided with a heating chamber (with temperatures between −70 °C and +250 °C) and a video extensometer for high-precision measuring of strain values. The tests were performed at room temperature (temperature values of 22 °C ± 1 °C and humidity of 35–45%) and at +39 °C. The selection of this temperature value for mechanical testing was made following both the “EASA Operations Manual example for UAS operations at SAIL II” [39], which specifies the temperature domain for civil unmanned aerial systems, as well as MIL-STD-810G [40], which specifies the general climatic conditions of the globe and the daily cycles of temperature, solar radiation, and relative humidity associated with them and the maximum values of parameters for operating conditions of unmanned aerial systems in standard climatic conditions and the intermediate daily cycle (Climatic Design Type: Basic, Daily Cycle: Intermediate-A3). Three-point bending tests were performed according to the ISO 178 standard [36], using a test speed of 2 mm/min, conventional deflection, conventional span length, and rectangular specimens, testing a minimum of 3 specimens/sample. Tensile tests were performed according to the ISO 527 standard [34], using type 1B dog-bone-shaped lab specimens with a test speed of 5 mm/min. In this case, also, a minimum of three specimens per sample were tested. In all mechanical testing, the data were analyzed by calculating the average for each type of sample, together with the standard deviation, and mechanical behavior was observed, illustrating the stress–strain curves of the mediated values.

**An impact test** was conducted at room temperature according to the Charpy ISO 179 A standard [35] (on unnotched specimens), using a 2J pendulum, angle: 150°, and a minimum of 4 test specimens with the following sizes: length: 10 cm, thickness: 4 mm, and width: 8 mm. Impact tests were performed using CEAST 9050 equipment produced by INSTRON, in Pianezza, Torino, Italy. At least 4 experiments were conducted for each composite specimen and average values were presented on the graphs. Red segments on the graphs are error bars.

**The dynamic contact angle** was measured using a VGA Optima XE system, AST Products, Billerica, MA, USA, equipped with dedicated software, by the sessile drop contact angle technique. This measurement offers important information regarding the hydrophobic/hydrophilic nature of a material, estimating the surface–liquid interaction type. The testing liquid was distilled water, using a drop volume of 5 µL for each sample and a 5 min interaction time between the liquid and sample. Measurements were performed in duplicate for each sample, recording 300 frames. VGA Optima Dynamic software version number 2.1.0.1. calculations and processing of the obtained frames were used to determine the average value of the contact angle for each sample.

**The surface free energy**, or surface tension, of solids may not be measured directly because of the elastic and viscous restraints of the bulk phase. This necessitates the use of indirect methods. One common method is contact angle measurement. A liquid “sessile” drop on a substrate surface will create a specific contact (tangent) angle at the solid, liquid, and air interface based on the surface tensions of the media. Consequently, a contact angle is the result of the interfacial surface tensions of these three phases and, with known fluid surface tensions, can be used to determine substrate surface energy. Surface energy is the sum of the various molecular forces. These molecular forces include dispersion energy, dipole–dipole energy, inductive energy, hydrogen bonding, and acid–base interactions. Dispersive forces exist between all molecules; however, other forces exist only when polar groups are present. Therefore, surface energy is most commonly represented by two terms, dispersive and polar.

**Thermal behavior** was followed with an STA 449C Jupiter system, TG-DSC (thermogravimetry–differential scanning calorimetry), from Netzsch (NETZSCH-Gerätebau GmbH, Selb, Germany). The sample (~10 mg) was inserted in an open crucible made of alumina and heated with 10 K·min^−1^ between 20 and 900 °C, in a dynamic (50 mL/min) air atmosphere.

**The FTIR maps** of the composite samples were recorded with a Nicolet iN10 MX (Nicolet, Waltham, MA, USA) FTIR microscope in domain 4000–600 cm^−1^ with a resolution of 8 cm^−1^.

**A fractographic analysis** was conducted, corroborating analysis results from both scanning electron microscopy techniques and optical microscopy. Even if a traditional optical microscope, unlike SEM, does not have a sufficiently extended focus depth, making it difficult to obtain focused images simultaneously for the peaks and troughs of broken surfaces [41], the use of optical systems coupled with modern specialized software greatly contributes to using the easy-to-process optical microscopy to perform a correct sample selection to be further analyzed via the expensive electron microscopy techniques [42].

**Optical microscopy** was performed in the fracture cross-section following tensile tests, at the 40× magnification level, using a Meiji ML 8520 optical microscope, Tokyo, Japan equipped with an Infinite Analyze-Lumenera Corporation video camera, Ottawa, ON, Canada for electronic image capture and recording. The analysis is intended to provide an overall image of the fracture mechanism involved in the tensile material failure, as well as identify the presence of voids and defects on an extended area of the cross-section as well as support sample selection to be subjected to the SEM analysis.

**Morphostructural characterization** was conducted on the as-received and surface-modified aramid fibers as well as in the fracture cross-section of the tensile-tested specimens. Micrographs were captured using scanning electron microscope (SEM) QUANTA FEI 250 equipment FEI/ThermoScientific, Eindhoven, The Netherlands, with a field emission gun with a resolution of 1.0 nm, using the secondary electron mode, while an elemental analysis was performed using its energy-dispersive X-ray spectroscopy (EDS) module. Given their non-conductive nature, before the analysis, the samples were plated with gold C7620 Mini Sputter Coater Quorum System, Laughton, East Sussex, UK.

**Elemental mapping analyses** were conducted using a high-resolution electron microscope (SEM) equipped with a field emission source (FEI Inspect F50, Eindhven, The Netherlands) at 30 kV and coupled with energy-dispersive X-ray spectroscopy (EDS).

## 3. Results and Discussion

### 3.1. FTIR Analysis of the Surface of Raw and SiO_2_-Modified Aramid Fibers

**Figure 9:**
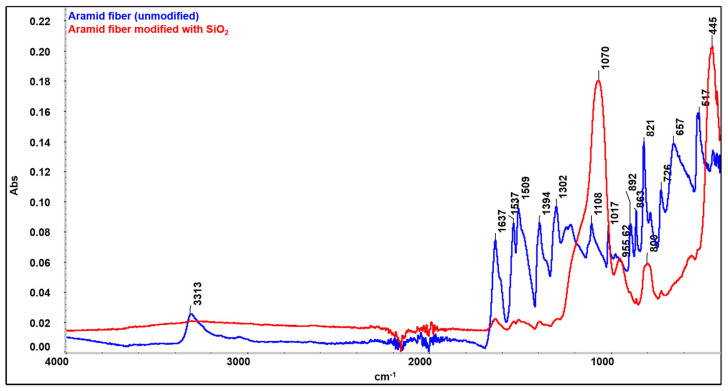
Overlapping spectra of raw aramid fibers (blue) and fibers modified with SiO_2_ (red).

**Figure 10:**
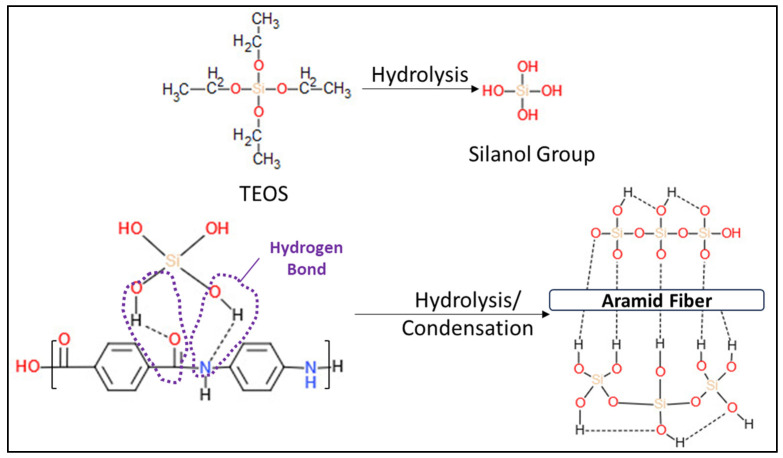
The proposed mechanism of the reaction between TEOS and the para-aramid fiber.

FTIR spectra obtained for raw (unmodified) aramid fibers and after modification with a precursor to form SiO_2_ particles are presented in Figure 9. The spectrum obtained for the raw (unmodified) aramid fiber shows characteristic functional groups from the fiber. The stretching band from the N-H bond at 3313 cm^−1^ (coming from the state of association of hydrogen bonds), namely the peak at 1637 cm^−1^, can be attributed to the stretching vibration of the -C=O group known as the Amide I band. As there is no separate peak in the proximity of the Amide I band, and from the analysis of the shape of the Amide I peak (with a shoulder), it is deduced that the peak due to the in-plane vibration of the -C=C- bond is overlapping with the Amide I band. Such conjugation is possible only in the 1,4-substitution of the benzene ring (para-substitution) [43]. The band at 1537 cm^−1^ (attributed to the bending vibration of the -N-H bond) and the one at 1302 cm^−1^ are due to the stretching vibration of the C-N bond, the in-plane bending vibration of the N-H bond, and the stretching vibration of the C-C bond specific to the Amide III band [44,45]. The band at 1509 cm^−1^ (Amide II) is caused by the stretching vibration of the C=C bond from the benzene ring skeletal structure. The peak at 1108 cm^−1^ corresponds to the in-plane deformation mode of the -C-H bond, and the sharp peak at 821 cm^−1^ corresponds to the out-of-plane deformation mode of the -C-H bond due to the two free hydrogen atoms, confirming once more the para-substitution mode occurring in the benzene ring cycle [43]. The band at 1394 cm^−1^ is due to semicircle stretching of free hydrogen of the aromatic ring. The band at 1017 cm^−1^ comes from the vibration of the C-H bond existing in the structure of the benzene ring [46]. The bands at 892 cm^−1^ and 726 cm^−1^ are the out-of-plane deformation modes of the N-H bond [47]. Comparing the spectrum obtained for the unmodified aramid fiber with the spectrum obtained for the modified fiber, new adsorption bands can be seen, at 1070 cm^−1^, 800 cm^−1^, and 445 cm^−1^, which can be associated with symmetrical and asymmetrical stretching vibrations of Si-O-Si bonds [33,48]. The band at 955.62 cm^−1^ can be associated with the symmetric vibration of the Si-OH (silanol) group [48]. The identification of these functional groups on the fiber surface confirms the synthesis of SiO_2_. It is commonly known that the Si-OH functional groups play a significant role in establishing interactions through hydrogen bonds with the functional groups that exist in the aramid fiber structure [49,50,51]. Moreover, it is noticed that bands originating from the structure of the aramid fiber disappear or decrease in intensity, which shows that they are covered/interact with SiO_2_ particles. Similar to the observations made by Zhou et al. [52], the reduction in intensity of the peak characteristic to the N-H group at 3313 cm^−1^ confirms the formation of an intermolecular hydrogen bond between the Si-O group in silica and N-H group in the aramid fiber structure. The proposed reaction mechanism, highlighting the formation of hydrogen bonds between the functional groups in the structure of the aramid fiber (especially N-H and C=O) and the groups derived from SiO_2_, is illustrated in Figure 10.

### 3.2. SEM and EDS Analysis of Raw and SiO_2_-Modified Aramid Fibers

**Figure 11:**
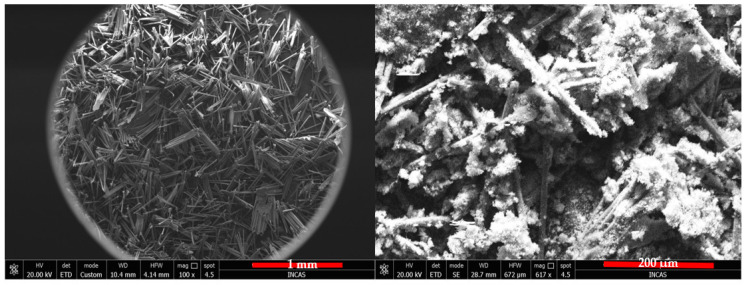
SEM micrographs of unmodified aramid fibers at 100× magnification level (**left**) and modified ones at 617× magnification level (**right**).

**Figure 12:**
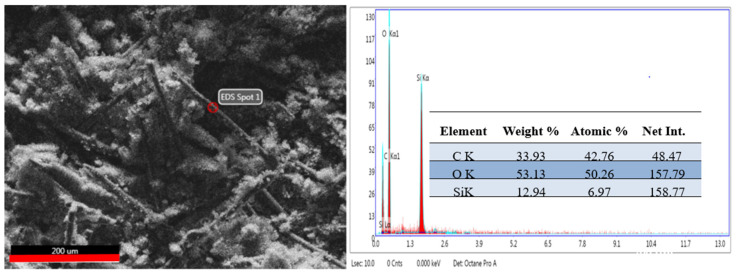
An SEM image of the SiO_2_-modified aramid fiber in the spot where EDS assessment was conducted (**left**) and the EDS spectrum and table resulting from the assessment in the marked spot (**right**).

SEM microscopy was used to study the morphology of the aramid fiber surface before and after modification/coating with SiO_2_ particles. Images obtained are presented in Figure 11 (left and right). In the case of unmodified aramid fibers, it is noticed that their surface is smooth and clean. This smooth surface of the fibers and implicitly their chemical inertia are due to the dense network of the surface, high axial orientation, and their rod-shaped structure [53]. Moreover, it can be noticed that unmodified aramid fibers present themselves as bundles of fibers resulting from the association of several individual fibers. This association is due to the adhesive applied by the producer, in order to protect their surface. In the case of aramid fibers modified with SiO_2_ particles using the sol–gel method, Figure 11 (right image), a dense and uniform coverage of their surface is noticed. The shape of SiO_2_ particles deposited onto the surface of fibers is spherical (with mesoporous structure) and at first glance they seem to be uniform in size. This increase in SiO_2_ particles on the surface of aramid fibers can be attributed to the hydrolysis of tetraethyl orthosilicate and subsequent condensation of a SiO_2_ oligomer in the sol–gel process, demonstrating that the chosen modification methodology was successfully performed [33,54]. The EDS analysis confirms once more the deposition of SiO_2_ particles onto the surface of aramid fibers. The EDS analysis was performed in the spot marked in the SEM image (Figure 12, left image) in an area where SiO_2_ particles are distributed as uniformly as possible, and the EDS spectrum and the table resulting from the analysis are presented in Figure 12, right image. As a result of the EDS analysis conducted on the surface of modified aramid fibers, the following elements were identified: C, O, and Si. According to the work published by Pelin et al. [55], the atomic ratio between O and C elements is 0.18 in the case of unmodified aramid fibers, while in the case of SiO_2_-modified fibers (obtained in the present study), it is higher than ~1.18. The increased content of O and Si can be associated with the generated Si-O-Si bond, caused by hydrolysis of the TEOS precursor, proving once more that the sol–gel technique is an efficient methodology in terms of modifying the surface of aramid fibers with SiO_2_. Results obtained are in good accordance with studies conducted by Lu Z et al. [53] (in the case of modifying aramid fibers pretreated with phosphoric acid followed by the sol–gel method, in the presence of TEOS), and the work published by Zang L et al. [33], in which aramid fibers were modified with SiO_2_ by bubbling in CO_2_, obtaining different atomic ratios between Si and C elements depending on the applied pressure (0.07%—10 MPa, 0.2%—12 MPa, 0.23%—14 MPa, 0.06%—16 MPa). In the present study, the atomic ratio between Si and C elements calculated following the modification of aramid fibers with SiO_2_ was ~0.16%.

**Figure 13:**
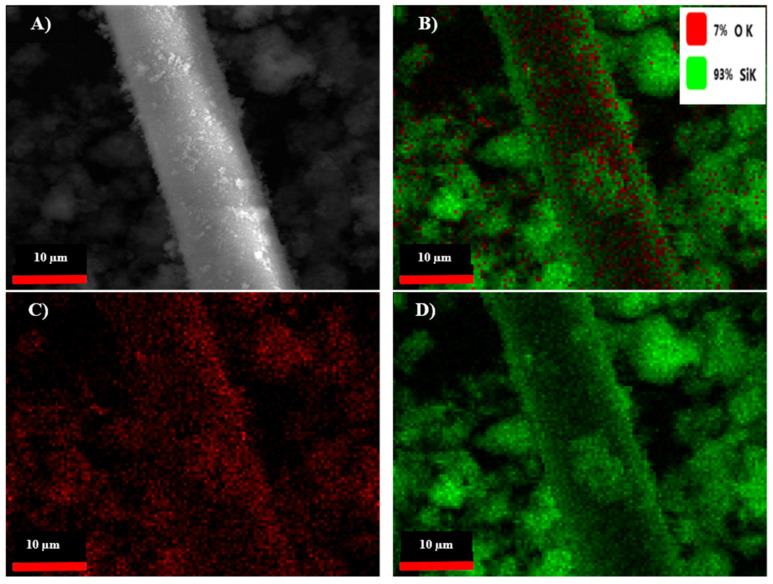
SEM micrograph (**A**) and EDS elemental mapping of constituent elements of SiO_2_-modified aramid fiber ((**B**)—obtained by overlapping O and Si elements); O and Si individual distribution in sample (**C**,**D**). All images were captured at 5000× magnification level.

For a better highlighting of the SiO_2_ particles’ distribution on the surface of the aramid fibers, a more detailed microstructure analysis was performed using SEM/EDS elemental mapping. Both SEM images and EDS maps were captured at 5000× magnification levels. Comparing with the SEM micrograph captured at a lower magnification level (Figure 11), the micrograph captured at 5000× (Figure 13A) shows a continuous deposition of SiO_2_ on the entire surface of the aramid fiber, with some limited agglomeration areas. The aramid fiber diameter measures approximately 13.3 µm. The EDS analysis illustrated in Figure 13B,D confirms the homogenous distribution of oxygen and silicium elements on the entire visualized area in the fiber, without attesting the presence of discontinuities (voids).

### 3.3. Assessment of Physical–Mechanical Properties of Composite Materials Reinforced with Aramid Fibers

#### 3.3.1. Tensile Behavior

**Figure 14:**
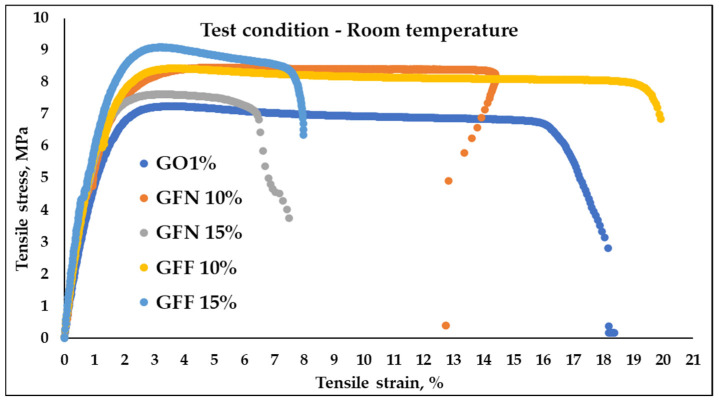
Tensile stress–strain curves collected at room temperature on mixtures: GO1% (control sample), GFN 10% and GFN 15% (mixtures containing 10 and 15 wt% unmodified aramid fibers), and GFF 10% and GFF 15% (mixtures containing 10 and 15 wt% SiO_2_-modified aramid fibers).

**Figure 15:**
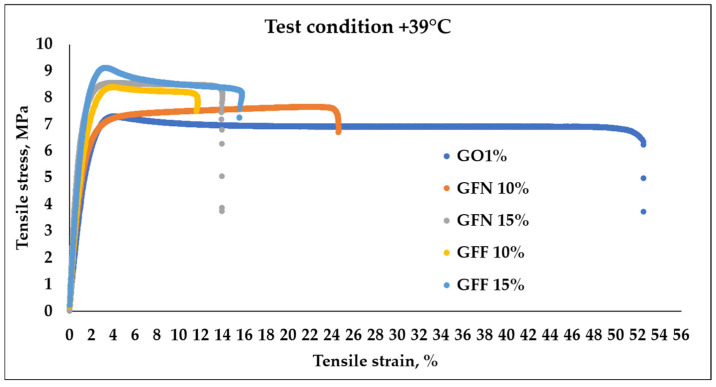
Stress–strain curves of mediated results obtained during tensile testing of the composites at +39 °C.

Typical tensile stress–strain curves of control samples GO1% and composites based on PP/PE-g-MA reinforced with 10 and 15% unmodified aramid fibers (GFN 10% and GFN 15%) and fibers modified with SiO_2_ (GFF 10% and GFF 15%), tested at room temperature and at 39 °C, are presented in Figure 14 and Figure 15. Values of flexural strength, modulus, and strain calculated from these curves are presented in Table 4. Analyzing the stress–strain curves obtained at room temperature, Figure 14, it is noticed that the initial slope of the curve is higher in the case of composites compared to the control sample, which reflects a higher tensile modulus (associated with the elastic deformation area, which occurs due to the stretching of atomic bonds); then, with increasing stress, the material leaves the elastic area and shows a behavior typical for ductile polymers, with a peak in the curve corresponding to the initiation of necking (yielding). Once the yield point is passed, the material will undergo permanent deformation (plastic deformation area) where atomic bonds break and realign. Subsequently, a reduction in stress and a leveling of the curve are noticed as the strain increases, which corresponds to the propagation and tracing of the neck region, followed by the breaking of the specimen. The stress–strain curve in Figure 14 shows that the GO1% sample fractured at a strain of 18.36 ± 3.26% while samples containing 10% aramid fibers (mixtures GFF 10% and GFN 10%) were fractured at a strain of ~19.87% and ~12.74. On the other hand, in the case of mixtures containing 15% unmodified/modified aramid fibers (GFN 15% and GFF 15%), a reduction in strain is noticed at ~7.5% and ~7.98% compared to samples GO1%, GFN 10%, and GFF 10%, which indicates that the fracture has become more fragile [56]. This is due to the presence of aramid fibers that make the composites more rigid and less deformable [57]. Compared to the control sample GO1%, mixtures containing unmodified/modified aramid fibers show higher values for the modulus and tensile strength, but lower elongation at break values, similar to findings by other authors [57]. In general, tensile strength is strongly dependent on the interaction occurring at the fiber/matrix interface while the modulus is less dependent, because it is calculated at lower strain values. In the case of the modulus, significant increases were noticed, linearly correlated to the addition of a higher amount of aramid fibers and implicitly to the stiffening effect due to their presence. The higher intrinsic rigidity of the embedded reinforcing agent prevents the mobility of polymer chains, leading to higher values in the case of composites compared to the control sample. Compared to other mechanical properties, Young’s modulus is not affected by the fiber/matrix interface, being rather the result of three main factors: the intrinsic modulus of the polymer, that of the reinforcing agent, and the amount of the embedded reinforcing agent [58]. Comparing results obtained in this study with similar materials existing in the literature that are based on PP and with similar percentages of aramid fibers (10–20 wt%), it is noticed that tensile strength and the modulus have lower values [59,60]. Considering that materials developed in this study are mainly meant for subsequent processing by thermoforming, where certain technological conditions are required, it is very difficult to make comparisons with existing data in the literature where materials based on PP reinforced with similar percentages of aramid fibers are intended for processing by injection and extrusion. Therefore, the matrix properties have a decisive influence on tensile strength of composites, and the addition of a coupling agent (PE-g-MA) to the composition induces ductility to polypropylene, which is reflected in a lower tensile strength and modulus, compared to those that do not contain a compatibilization agent, and the addition of fibers cannot compensate for this. Tensile strength relative to the control sample GO1% improved by 17% (in the case of samples GFN 10% and GFF 10%) and by 19 and 26%, respectively (GFN 15% and GFF 15%). The highest tensile strength values were obtained for the mixture containing 15% modified aramid fibers (the GFF 15% mixture), which proves that the SiO_2_ particles deposited can improve the roughness of the surface and can effectively prevent the fiber from being pulled out of the matrix. Therefore, deposition is an efficient method for fiber modification, with advantages brought to the fiber structure, such as repairing surface defects and high compatibility with different types of matrices [61]. Similar results were obtained in the case of MWCNT anchoring to the surface of the p-aramid fiber (p-AF) used as a reinforcing agent in vinyl ester resin (VE), contributing to an increased roughness of the fiber surface, to mechanical interlocking between the fiber and matrix, and to friction at the fiber/matrix interface without damaging the fiber. As a result, the tensile and flexural properties of composites p-AF/VE were, also, improved [62]. However, there are no remarkable increases in the mechanical properties of mixtures containing modified aramid fibers compared to mixtures containing unmodified aramid fibers. This can be attributed to the fact that the sizes of SiO_2_ particles deposited onto the surface of aramid fibers are micrometric, and in this case the formation of stable interfacial hydrogen bonds between fiber and SiO_2_ is lower than if the particle size fell within the nanometer range (the smaller the particle radius, the more the energy density of the hydrogen bond and interfacial interaction improves, thus enhancing the stability of the interface) [63]. There is also the possibility that between the maleic anhydride groups located on the surface of the PE polymer chain, there may be interactions through hydrogen bonds with hydroxyl groups present on the surface of aramid fiber—SiOH, similar to the case of using natural fibers [58] or artificial marble (CaCO_3_) [64].

The stress–strain curves obtained for the control sample (GO1%) and for composites reinforced with unmodified/modified aramid fibers, obtained at the temperature of 39 °C, are presented in Figure 15 and values calculated from curves are presented in Table 4. The resulting curves show a similar behavior to mixtures tested at room temperature with the specification that strain increases with the temperature, while the modulus and tensile strength significantly decrease. In general, it was noticed that tensile strength and the modulus decrease with the increase in temperature, while strain at fracturing increases with the increase in temperature (because the mobility of the molecular chain intensifies). This effect was found to be more pronounced in unreinforced materials than in materials containing reinforcing agents [65]. Therefore, the highest strain variation is obtained in the case of the control sample (GO1%) tested at 39 °C with increases of 187% relative to the value obtained at room temperature. Mixtures containing unmodified and modified aramid fibers show strain increases below 100% compared to values obtained at room temperature. In all cases, the value of the modulus and tensile strength decreases for mixtures tested at 39 °C compared to the same mixtures but tested at RT (room temperature). This changing trend was due to molecular effects of the polymer matrix and fiber. With the increase in temperature, the molecular energy increased and thus the rotation, vibration, and motion increased as well. Thus, the molecular distance was increased with the increase in temperature, which leads to lower values of the modules and tensile strength, similar to results obtained in the case of PP/Kevlar fiber composites tested at the temperature of 30 and 50 °C [66].

#### 3.3.2. Flexural Behavior

**Figure 16:**
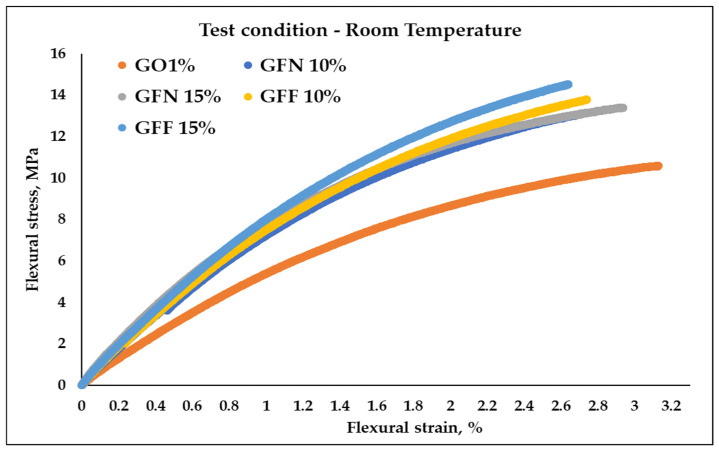
Stress–strain curves of mediated results obtained during flexural testing of the composites at room temperature.

**Figure 17:**
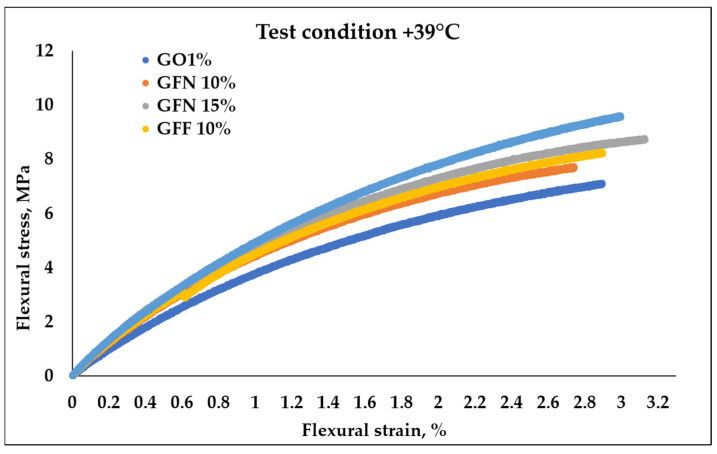
Stress–strain curves of mediated results obtained during flexural testing of the composites at +39 °C.

Figure 16 and Figure 17 present the stress–strain curves obtained as a result of 3-point flexural tests, at room temperature and at 39 °C, for mixtures GO1% (50/50 PP K948/PE-g-MA and 1 wt% Mastersafe MP-10-20B gray pigment), for mixtures GFN 10% and GFN 15% (with the same basic composition as GO1% with the mention that they contain different percentages of unmodified aramid fibers and 5 wt% wax based on polyethylene), and for mixtures GFF 10% and GFF 15% (similar to GFN 10% and GFN 15% with the mention that aramid fibers were modified with SiO_2_). Results from flexural tests at RT (Table 5) are similar to those from tensile strength tests, obtaining higher values in the case of composites reinforced with fibers compared to the control sample (GO1%). Typical bending stress–strain graphs of samples tested at room temperature, Figure 16, show the transition of samples from elastic deformation (this area appears due to the changes in the lengths and bond angles of the macromolecules, the magnitude of the deformation being proportional to the magnitude of the external force; the stress–strain relationship is linear and respects Hooke’s law) to plastic deformation (viscoplastic behavior), demonstrating that tested materials show ductile behavior [67]. The curves show that the value of the flexural strength increases with the increase in the deformation rate (strain), in the case of all the tested samples. From the analysis of the obtained curves, it is found that the composite materials developed in this work did not fracture after the flexural test, similar to the results obtained in the case of composites based on 90 wt%/10 wt% (PP/aramid fibers) [68]. Compared to tensile testing, flexural testing was not conducted until fracturing, but until the conventional deflection, as recommended by a standard for ductile materials. Flexural strength increases linearly with the increase in aramid fiber content, as shown in Table 5 from 10.62 MPa obtained for the control sample (GO1%) to 13.8, 13.43, 13.81, and 14.54 MPa in the case of composites (GFN 10%, GFN 15%, GFF 10%, and GFF 15%). The flexural modulus follows a similar trend to flexural strength, with increases of 40, 62, 41, and 53% obtained for mixtures GFN 10%, GFN 15%, GFF 10%, and GFF 15% compared to GO1%. The increase in fiber content that exhibits a brittle nature generates a reduction in the mobility of the ductile polymer molecules, therefore leading to materials with lower flexural strain, compared to the control sample. The highest flexural strength values were obtained in the case of mixture GFF 15% with increases of 37% and 8%, respectively, relative to mixtures GO1% and GFN 15%. Therefore, it can be concluded that the applied stress is transferred from the matrix by means of aramid fibers modified on the surface with SiO_2_, resulting in improved mechanical properties. By means of the maleic anhydride group existing on the PE surface, the interfacial resistance is improved, because the agglomeration of the aramid fibers is limited, resulting in a greater degree of dispersion in the polypropylene matrix. This improvement in interfacial adhesion occurs because during the preparation of the composites, the -OH groups present on the surface of the SiO_2_ particles react through a nucleophilic mechanism with the maleic anhydride groups (possibly chemically), which prevents agglomeration. Also, polar interactions, such as those due to hydrogen bonding, are more likely to occur between the carboxyl groups of the grafting agent and the surface hydroxyl groups of the silica molecules. All these mechanisms listed above could improve the compatibility between the polymer matrix and SiO_2_ by improving the degree of dispersion and the adhesion between the phases [69,70]. Moreover, it was found that the presence of nanostructures on the surface of the fibers increase the stress transferability at the interface, indicating that the primary adhesion mechanism between the fiber and the polymer matrix occurs through mechanical anchoring [71,72]. The existing studies in the specialized literature show higher values of flexural strength (43.1 ± 3.3, 49.8 ± 2.1, 48.8 ± 0.5, 33.3 ± 1.2, and 12.2 ± 1.9 MPa) and the modulus (1.10 ± 0.02, 1.36 ± 0.03, 1.71 ± 0.02, 1.5 ± 0.02, and 1.35 ± 0.02 GPA) in the case of composites based on PP reinforced with 10, 20, 30, 40, and 50 wt% discontinuous aramid fibers [73] and higher values of flexural strength (38, 41.98, 46.58, and 53.79 MPa) in the case of PP composites reinforced with 0, 10, 20, and 30 wt% aramid fibers [60], compared to the values obtained in this study. These higher increases can be attributed primarily to the properties of the PP matrix, which show higher values of the flexural strength, ~38 MPa, compared to the value of 10.62 ± 0.17 MPa obtained in the case of the GO1% control sample.

Measurements at 39 °C (Figure 17 and Table 5) indicated an increase by 9–23% for unmodified fibers and 16–35% for modified ones in terms of strength, and by 24–35% for unmodified fibers and 28–33% for modified ones in terms of the modulus, compared to the control sample. It can be observed that flexural strength and the modulus showed lower values when the materials were tested at higher temperature. This could be due to the higher capacity to deform generated by the thermoplastic chains’ freedom of movement increase with a temperature increase, which consequently generates less resistance of the material when subjected to loadings in bending, similar to observations by other researchers in the case of tests performed at 25, 40, 60, 80, and 100 °C [67].

#### 3.3.3. Impact Strength

**Figure 18:**
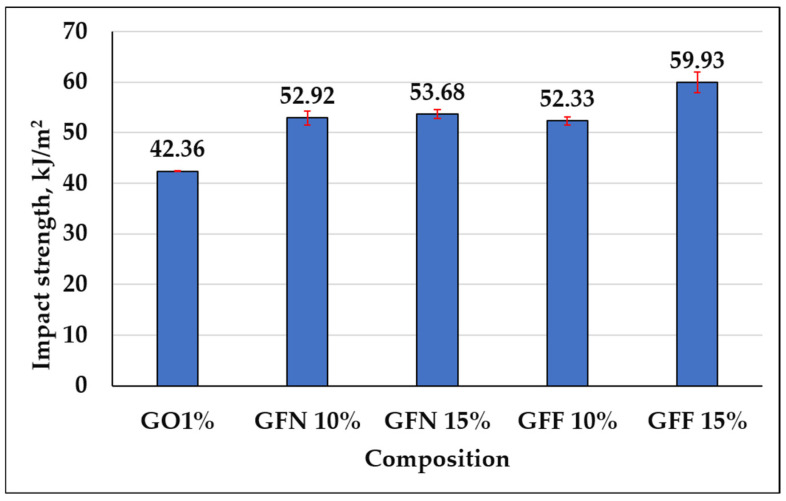
Impact strength for GO1% (control sample), mixtures containing unmodified aramid fibers (GFN 10% and GFN 15%), and mixtures reinforced with SiO_2_-modified aramid fibers (GFF 10% and GFF 15%).

Impact strength for the control sample and mixtures containing unmodified and SiO_2_-modified aramid fibers is presented in Figure 18. Impact strength improves by 25–27% in the case of composites containing unmodified aramid fibers and by 24–42% in composites containing SiO_2_-modified aramid fibers compared to the control sample (GO1%). These increases may be due to a more effective transfer between the matrix and aramid fibers. Given the interaction between PE-g-MA and PP taking place through the entanglement of molecular chains or co-crystallization, the generation of microcracks during impact testing is inhibited, the matrix having a higher capacity to absorb a great part of the energy (due to its ductile nature) and to efficiently transfer it through the interface to the aramid fibers, which leads to higher impact strength values [64]. Compared to existing studies in the literature based on similar composites (PP reinforced with hydrolyzed and microfibrillated Kevlar fibers) [74], PP reinforced with 10, 20, and 30 wt% aramid fibers [60] and PP reinforced with different percentages of 10, 20, 30, 40, and 50 wt% short aramid fibers [73], impacting strength values obtained in this study are clearly superior, due to the presence of the compatibilizer, which induces flexibility to the matrix and ensures an efficient transfer through the fiber interface.

#### 3.3.4. Analysis of Water Contact Angle and Surface Energy Obtained on Composites Reinforced with Aramid Fibers

**Figure 19:**
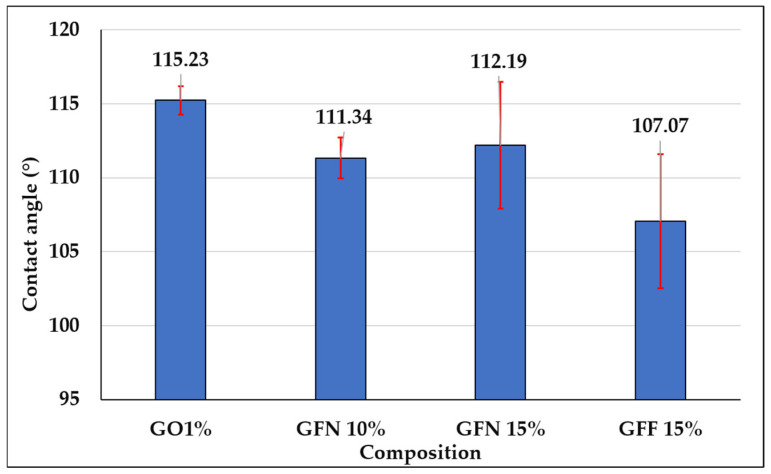
Water contact angle values of mixtures: GO1% (control sample), GFN 10% and GFN 15% (samples based on PP/PE-g-MA reinforced with unmodified aramid fibers), and GFF 10% and GFF 15% (mixtures containing SiO_2_-modified aramid fibers), respectively.

**Figure 20:**
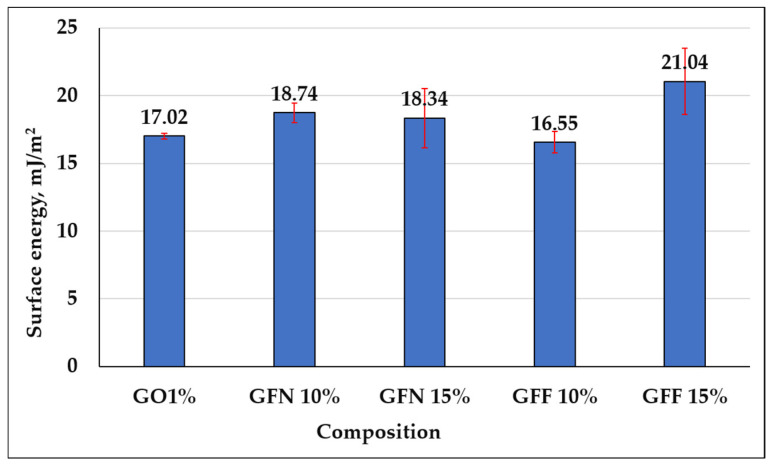
Surface energy calculated for mixtures: GO1% (control sample), GFN 10% and GFN 15% (samples based on PP/PE-g-MA reinforced with unmodified aramid fibers), and GFF 10% and GFF 15% (containing SiO_2_-modified aramid fibers).

**Figure 21:**
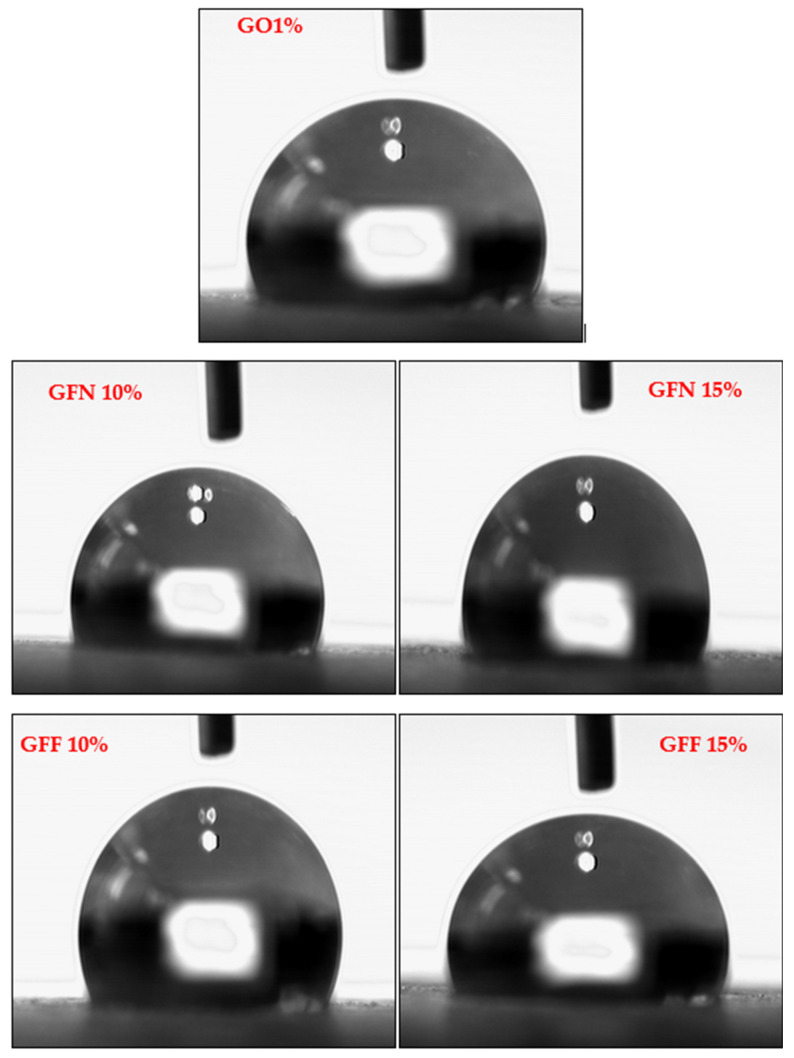
Images of water drop–solid surface contact angle obtained for control sample (GO1%); samples with polymer mixture (PP/PE-g-MA) as matrix, containing 10 and 15% unmodified aramid fibers (GFN 10% and GFN 15%); and PP/PE-g-MA samples reinforced with 10 and 15% SiO_2_-modified aramid fibers (GFF 10% and GFF 15%).

Contact angle determination is an important parameter that can be used to describe the hydrophobicity of a surface and its resistance to wetting. Results of dynamic contact angle measurements for the five samples tested in contact with a polar liquid (water), depending on the composition of mixtures, are presented in Figure 19, the calculated energy is presented in Figure 20 and representative images for water drop-sample surface contact are presented in Figure 21. The red segments marked on Figure 19 are error bars and values presented on the graph were calculated as the average of two measurements/sample. Based on obtained values, it can be concluded that all tested mixtures are hydrophobic with a contact angle value of >90°. However, depending on the composition of mixtures, the contact angle value varies by a maximum of ~8°, in the sense that it decreases. In the case of the control sample (GO1%), the highest contact angle value of 115.23° is obtained if the error bar is taken into account. In the case of composites containing 10 and 15% unmodified aramid fibers (samples GFN 10% and GFN 15%), the contact angle value decreases with the higher fiber percentage. This can be attributed to the hydrophilic nature of aramid fibers, which allow for the specific interaction between water and the fiber surface, enabling it to penetrate the fibers, leading to their swelling, to a decrease in interfacial energy, and implicitly to a reduction in the contact angle [75]. According to the literature, the water contact angle of Twaron aramid fiber is 57.11 ± 3.79 [76]. In the case of composites containing SiO_2_-modified aramid fibers (GFF 15%), the contact angle value decreases compared to sample GO1% and sample GFN 15%, respectively. This may be due to the fact that the higher the amount of SiO_2_-modified aramid fibers, the higher the hydrophilicity of the mixture. This reduction is probably due to the fact that the higher percentage of added SiO_2_-modified aramid fibers may generate pores/surface defects in the polymer matrix or a lower compaction of the material. Through the orifices of these pores, water penetrates the material more easily and due to the open porous surface structure and to the presence of hydrophilic hydroxyl groups, moisture adsorption increases with the formation of hydrogen bonds, which leads to a reduction in the contact angle [77]. However, regardless of the tested composition, all composites have a hydrophobic nature, variations being influenced only by the composition. The surface energy was calculated for both control sample GO1% and for mixtures containing SiO_2_-modified aramid fibers, and results are shown in Figure 20. Based on these results, an increase in surface energy is noticed, particularly in mixture GFF 15%, which reflects a higher wettability degree of fibers with a polymer phase (due to the increase in the polar fraction). Therefore, the higher the surface energy (the polarity of the surface increases), the higher the adhesion between the phases implicitly, but the lower the contact angle values [78]. These findings are in very good accordance with results of mechanical tests, FTIR, SEM analysis, etc., and mixture GFF 15% presents optimal properties due to very good adhesion between the phases.

### 3.4. FTIR Analysis on Polymer Composites Reinforced with Aramid Fibers

**Figure 22:**
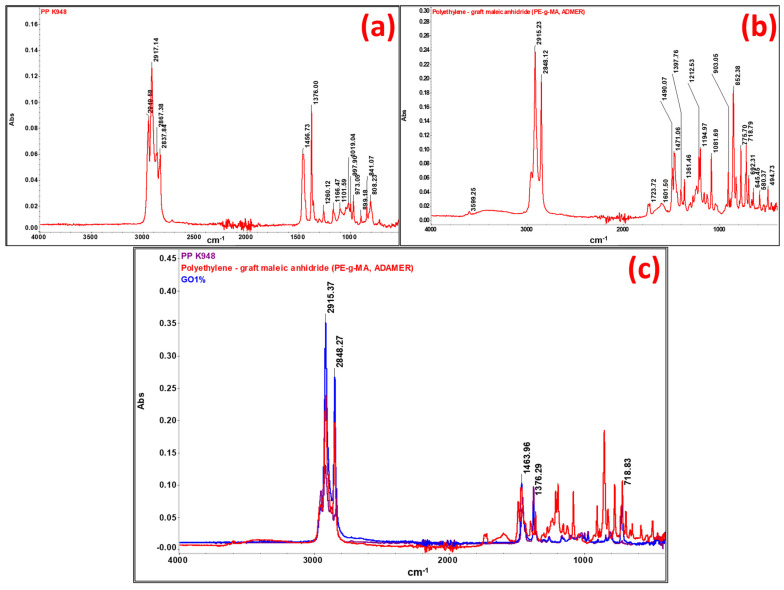
FTIR spectra for (**a**) PP K948, (**b**) PE-g-MA (ADMER), and (**c**) overlapping individual spectra of PP K948, PE-g-MA, and GO1% mixture (considered control sample containing 50:50 PP K948:PE-g-MA and 1 wt% Mastersafe gray pigment).

Fourier-transformed infrared spectroscopy (FTIR) was performed in order to highlight the interactions taking place between components (PP K948, PE-g-MA, aramid fibers, etc.). FTIR spectra were also used for raw materials used in developing composites, namely PP K948 (Figure 22a) and PE-g-MA (Figure 22b), to identify any interaction occurring between the components. The spectrum obtained for the control sample GO1% (containing 50:50 PP K948:PE-g-MA and 1 wt% Mastersafe gray pigment) in Figure 22c is presented superimposed with the spectra for PP K948 and PE-g-MA in order to highlight the formation or disappearance of bonds. The presence of the gray pigment based on aluminum does not lead to the appearance or disappearance of spectral bands, being identical to the spectrum obtained in the absence of the pigment and for this reason it has not been presented separately in this paper. The analysis of the spectrum obtained for the GO1% sample shows bands originating from PP K948 superimposed with the bands originating from PE-g-MA (mainly at 2915.37 and 2848.27 cm^−1^ with minor shifts relative to their position in individual components) and the band at 1463.96 cm^−1^ with shifts of ~7 cm^−1^ from its position in PP K948 and PE-g-MA, proving that weak interactions occur between the components; the peat at 1376.00 cm^−1^ originates from PP K948 and the one at 718.83 cm^−1^ originates from PE-g-MA. Based on obtained spectra, it can be concluded that the macromolecular chains from PP K948 and PE-g-MA have entangled or co-crystallized together [64]. The spectrum of polypropylene presented in Figure 22a highlights the functional groups existing in its structure. Thus, the band at 2949.58 cm^−1^ may be associated with the asymmetric stretching vibration of the CH_3_ bond, 2917.14 cm^−1^ with the asymmetric stretching vibration of the CH_2_ bond, 2867.38 cm^−1^ with the symmetric stretching of the CH_3_ bond, and 2837.84 cm^−1^ with the symmetric stretching vibration of the CH_2_ bond. The symmetric bending vibration of the CH_3_ bond can be highlighted at 1456.73 and 1376.00 cm^−1^, respectively. Vibrations at 1166.47 and 973.05 cm^−1^ come from the stretching vibrations of CH-CH_3_ groups, and the average intensity absorption peak at 841.07 cm^−1^ is due to the vibration of the C-H bond. The peak at 997.9 cm^−1^ (coming from the stretching and rocking vibration of C-C and CH_3_ bonds) and the band at 808.23 cm^−1^ come from the stretching vibration of the C-C bond [79,80,81]. The spectrum obtained for PE-g-MA in Figure 22b highlights the characteristic bands from polyethylene and maleic anhydride. Thus, the bands at 1723.72 and 1601.50 cm^−1^ are associated with the symmetric stretching vibration of the C=O group from the maleic anhydride grafted onto polyethylene [82]. The bands at 2848.12 and 2915.23 cm^−1^ correspond to symmetric and asymmetric stretching vibrations of the -C-H bond from ethylene fragments [83]. The bands at 1471.06 cm^−1^ (bending) and 718.79 cm^−1^ (rocking) come from the -CH_2_ group existing in the polyethylene skeleton [84]. The band at 1212.53 cm^−1^ may be associated with the C=C group from maleic anhydride [85].

**Figure 23:**
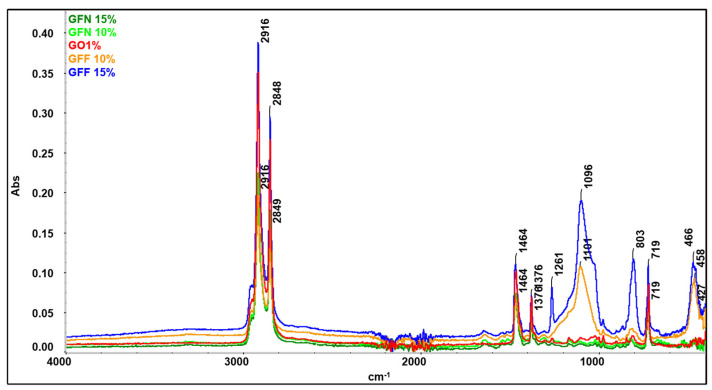
Overlapping FTIR spectra of the control sample (GO1%), mixtures containing unmodified aramid fibers (GFN 10% and GFN 15%), and mixtures containing SiO_2_-modified aramid fibers (GFF 10% and GFF 15%).

Spectra for mixtures GFN 10% and GFN 15% (Figure 23) overlap perfectly on the control sample GO1% (used as reference), the presence of fibers not being highlighted probably due to the fact that they are coated with a thick layer of a polymer phase [55]. On the other hand, for mixtures GFF 10% and GFF 15%, characteristic bands from functional groups from the aramid fiber cannot be detected, but the bands originating from SiO_2_ can be seen. It is noticed that the intensity of these bands increases in the case of mixture GFN 15% compared to GFF 10% as the amount of aramid fibers modified with SiO_2_ is higher. In the case of mixtures GFF 10% and GFF 15%, the position of bands associated with stretching and bending vibrations of Si-O-Si bands appears at 1101–1096 cm^−1^, 803 cm^−1^, and 458–466 cm^−1^, respectively [86,87]. On the other hand, in the case of the spectrum obtained for the aramid fiber modified with SiO_2_ (presented in another section of this paper, Figure 9), the bands originating from the Si-O-Si group appear at 1070, 800, and 445 cm^−1^. Therefore, there are major shifts of the position of bands to higher values, which proves that SiO_2_ present on the surface of aramid fibers strongly interacts with the maleic anhydride group in polyethylene [69]. In the case of the GFF 10% sample, the position of the band at 1101 cm^−1^ shifts by 31 cm^−1^ while the band at 458 cm^−1^ shifts by 13 cm^−1^ relative to their positions in the SiO_2_-modified aramid fiber. In the GFF 15% mixture, the position of the band at 1096 cm^−1^ shifts by 26 cm^−1^ while the band at 466 cm^−1^ shifts by 21 cm^−1^ relative to their positions in the SiO_2_-modified aramid fiber_._ Based on the FTIR analysis, improved physical–mechanical results are confirmed in the case of using SiO_2_-modified aramid fiber, due to a good adhesion between the phases, in accordance with SEM images, namely the contact angle/surface energy.

### 3.5. DSC-TG Analysis

**Figure 24:**
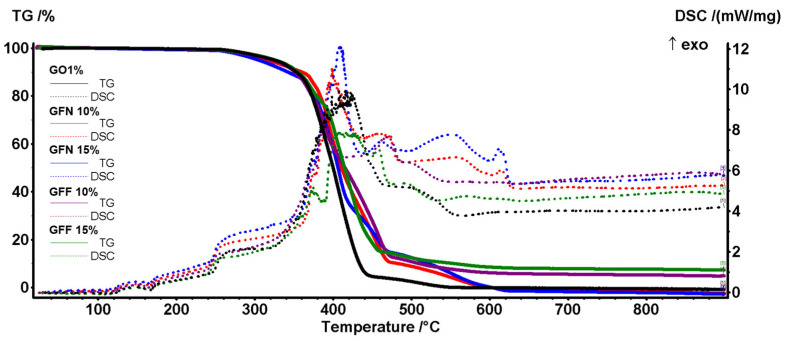
The TG-DSC curves for all five composite samples.

The thermal stability of the composite samples was assessed by the TG-DSC analysis (Figure 24). All five samples can be considered inert up to 250 °C, with mass loss of ~1% generated by the oxidation of additives from the pigment and superficial oxidation. The small endothermic effect from ~165 °C marks the end of the melting process and is generated by the polypropylene part of the composite. At higher temperatures, the polymer backbone starts to fragment and some of these moieties are quickly oxidized. Therefore, 5% mass loss is recorded for all samples between 304 and 327 °C, while 50% mass loss takes place between 400 and 416 °C as indicated by data from Table 6. Between 250 and 600 °C, the DSC curves are dominated by multiple overlapped exothermic effects indicating the oxidation of the broken organic fragments and total burning of the carbonaceous residual mass. The control sample GO1% is the first one reaching the end of the degradative–oxidative process. All samples containing aramid fibers are oxidized slower due to the aromatic nature of their structure, and therefore higher temperatures are needed to completely oxidize the GFN and GFF samples. For the GFN samples, a late exothermic effect, in the interval 550–560 °C, is related to the oxidation of the unprotected aramid fibers, the small peak from 610 to 615 °C representing the burning of residual carbon from aramid fibers. In the GFF samples, the aramid fibers are oxidized very slow at a high temperature due to the SiO_2_ protection, and therefore the exothermic effects are low in intensity over 500 °C. Additionally, for the samples containing the SiO_2_-modified aramid fibers, the residual mass is represented by the inorganic part, namely silica. The residual mass values permit an estimation of the SiO_2_ loading, of roughly ~50%, for the modified aramid fibers.

**Figure 25:**
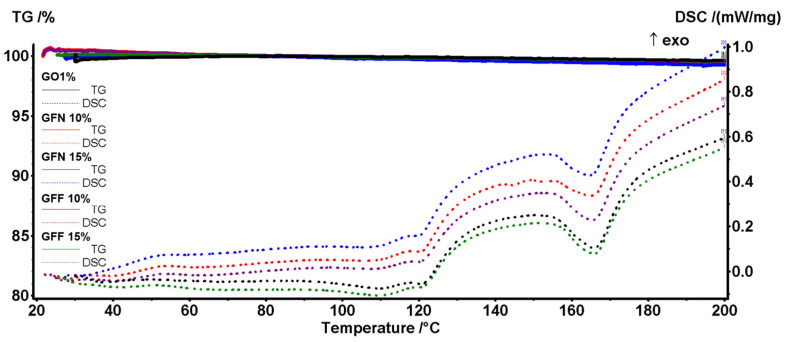
Zoom-in view of TG-DSC curves in 20–200 °C temperature interval for all five composite samples.

As the composites are used for objects that will operate in normal conditions, a closer look at the DSC peaks in the interval 20–200 °C will reveal that the introduction of the aramid fibers did not induce negative effects on the stability of the composites (Figure 25).

The melting onset for all samples is between 154 and 156 °C, with a higher value for the GFF samples containing the silica-modified aramid fibers. The presence of SiO_2_ acts as a stabilizing agent, which takes over part of heating energy from the rest of the sample. The principal data from the thermal analysis are presented in Table 6.

### 3.6. Optical Microscopy

Optical micrographs were captured in the cross-section of the samples after fracturing following tensile load testing at room temperature, providing information regarding the morphology of the samples and overall observations on fracture mechanisms, which will subsequently be corroborated with fractographic information offered by SEM.

**Figure 26:**
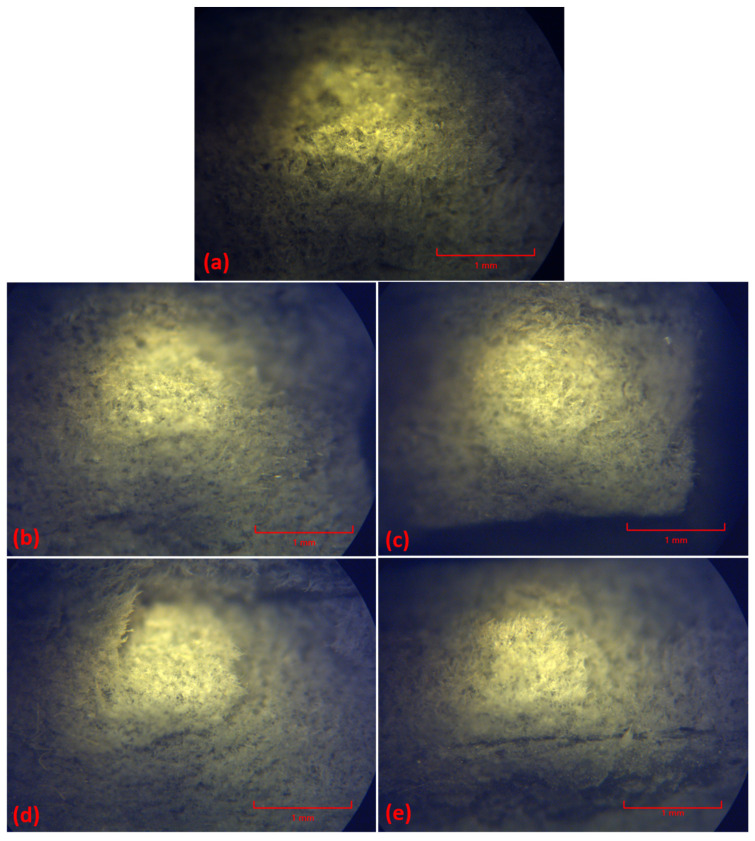
Optical micrographs of cross-section of (**a**) control sample GO1%, (**b**) KFN 10%, (**c**) KFN 15%, (**d**) KFF 10%, and (**e**) KFF 15%.

Figure 26 illustrates the fracture cross-section of one specimen of each sample fractured following tensile testing at room temperature. The control sample illustrated in Figure 26a presents a classic morphology, characteristic to a thermoplastic, ductile fracture, showing a compact structure, with a fibrous-like (sponge-like) appearance, which indicates an overall presence of voids uniformly distributed and owed to the structure appearance. When aramid fibers are present, the morphology becomes a denser one, increasing with fibers’ content, as shown in Figure 26b,d, illustrating samples with 10 and 15%, respectively, as-received aramid fibers added. While the control sample and samples containing 10% aramid fibers as-received (Figure 26b) or functionalized (Figure 26c) exhibit a cross-sectional fracture, almost perpendicular to the force application plane, it can be observed that samples containing 15% aramid fibers as-received (Figure 26d) or functionalized (Figure 26e) show a more brittle-like fracture, with some microcracks propagating, indicating the tearing of the material in different planes.

All samples show a uniform appearance over the entire visualized surface, and all micrographs support the observations obtained in FTIR spectroscopy, indicating the strong embedment of the fibers into the thermoplastic matrix due to a physical interlocking between them, leading to uniform morphologies, reduced surface defects, and consequently toughening and improving mechanical load transfer into the composite.

### 3.7. SEM Analysis

**Figure 27:**
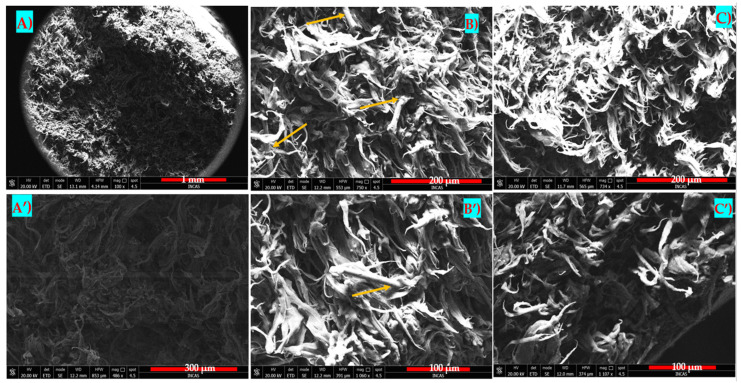
SEM images obtained for samples: GO1% (images (**A**,**A’**) at magnitudes of 100× and 486×), GFN 15% (images (**B**,**B’**) at magnitudes of 750× and 1060×), and GFF 15% (images (**C**,**C’**) at magnitudes of 734× and 1107×). Yellow arrows highlight the presence of aramid fibers.

SEM microscopy was performed in the fracture section as a result of tensile strength testing. SEM images provide valuable information regarding adhesiveness between the components, but also allow the visualization of the degree of dispersion and distribution of unmodified/modified aramid fibers in the PP/PE-g-MA matrix. Analyzing the fractured surface of the GO1% sample (Figure 27A,A’), a fibrillar morphology is noticed, and the presence of voids as well as the presence of polymer fibrils (that have elongated following the tensile strength test, undergoing permanent deformation), showing once more the ductile nature of the mixture, similar to findings from the analysis of stress–strain curves obtained after tensile/flexural testing [88]. No areas with phase separations were highlighted, which proves that the molecular chains of PP with PE-g-MA were entangled/co-crystallized [64]. In the case of the GFN 15% mixture (Figure 27B,B’), a denser and more compact morphology is noticed, and the presence of the aramid fibers as well as twisted polymer fibrils, proving that these fibrils first elongated and then broke. Analyzing the fractured ends of polymer fibrils, the ductile nature of the mixture is noticed. When analyzing the area where the presence of aramid fibers is obvious (marked with yellow arrows on the images), no areas covered with a polymer phase could be seen. However, there is a firm embedding of these in the polymer matrix (without the existence of clear outlines/voids at the fiber/matrix interface) (as is clear in image B’), proving that there are weak interactions between the components. In the case of sample GFF 15% and Figure 27C,C’, the presence of aramid fibers could not be highlighted. Either the aramid fibers are strongly anchored/fixed in the matrix, or they are completely covered with the polymer phase due to higher wettability and compatibility (results supported by FTIR analysis, mechanical tests, contact angle/surface energy) [80,89]. GFF 15% mixtures maintain their fibrillar structure, with some areas with porosities/voids in the material.

### 3.8. FTIR Microscopy

**Figure 28:**
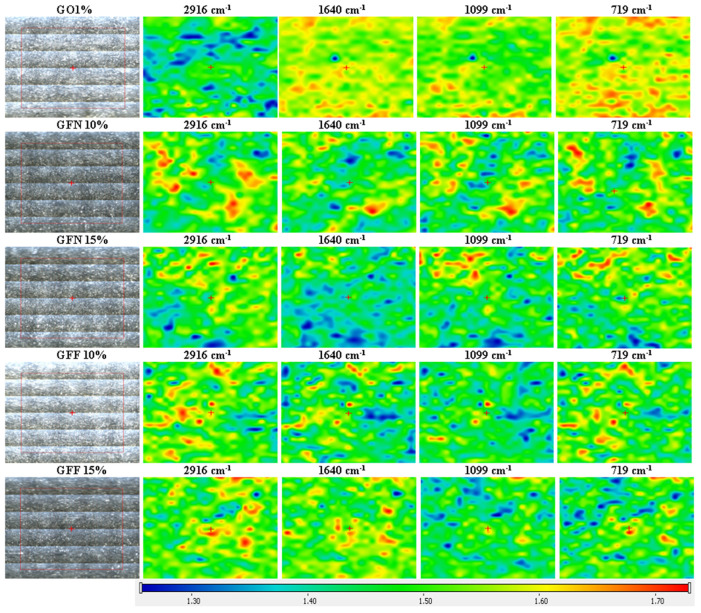
FTIR microscopy maps of composite films at 2916, 1640, 1099, and 719 cm^–1^ (red areas indicate the highest absorbance, while blue areas correspond to the lowest absorbance).

The FTIR microscopy maps recorded for multiple wavenumbers are presented in Figure 28. The selected wavenumbers are 2916 cm^−1^ for C-H asymmetric stretching vibration from CH_2_, 1640 cm^−1^ for the C=O stretching vibration from aramid fibers, 1100 cm^−1^ for the Si-O-Si vibration from SiO_2_ (but also fingerprint region, for stretching vibrations of the C–O bond), and 721 cm^−1^ for the rocking vibration of the methylene group [90]. As GO1% does not contain aramid fibers and SiO_2_, the FTIR maps at 1640 and 1099 cm^−1^ present almost no variation in the absorption intensity. The FTIR map from 719 cm^−1^ coupled with the one from 2916 cm^−1^ is a good indicator of the polyolefin distribution in the composite samples.

The introduction of aramid fibers in GFN samples does not alter their homogeny. The FTIR maps from 1640 and 1099 cm^−1^ are confirming the distribution pattern presented by the FTIR maps from 2916 and 719 cm^−1^ for both GFN and GFF samples. The functionalization of aramid fibers with SiO_2_ is leading to the dominance of the 1099 cm^−1^ band over 1640 cm^−1^, which is confirmed by the maps from 1099 cm^−1^ for GFF composite samples.

Overall, the FTIR maps indicate a good homogeneity of the composite samples, at the micrometer level.

### 3.9. Production of Cover for the Drone and Flight Test

The sheets developed based on the samples tested and characterized were vacuum-thermoformed following the procedure and parameters described. All developed sheets were successfully thermoformed onto the two specific shape molds that compose the cover for the drone’s body. The sheets were easily detached from the molds’ surface, but the ease to detach was better in the case of 3D-printed molds due to the rugosity of the surface of the molds.

**Figure 29:**
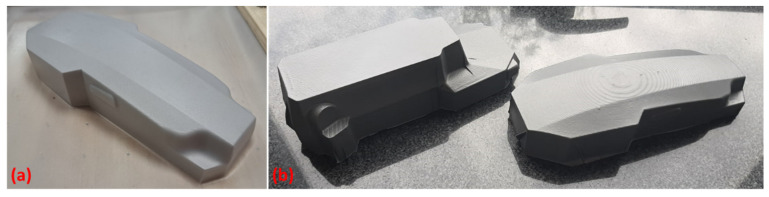
(**a**) Control sample GO1% thermoformed on upper CNC-machined mold. (**b**) GFF 10% thermoformed on upper and lower 3D-printed molds.

When analyzing the sheets thermoformed using the two types of molds, it can be observed that sheets thermoformed on the CNC machines’ molds exhibit a very smooth surface (Figure 29a), while the one thermoformed on the 3D-printed molds exhibits a pattern that reproduces the draglines on the mold’s surface, attributed to the filament overlapping during printing (Figure 29b). It is important to mention that these draglines could have been corrected through surface post-processing, but in this case were beneficial for adding supplementary rigidity to the cover. Provisioning this kind of dragline directly in the design would have added even more complexity to the CNC machining procedure as well as the 3D printing method, and therefore this feature of the printing was used as an advantageous one for our application.

After thermoforming, the two pieces that compose the cover were clipped to be mounted on the drone’s body (cutting supplemental areas, like the one covering the video camera and the ones where propeller supports are attached). It is important to mention that the two pieces of the cover were weighed before attaching them to the drone, adding a weight increase of about 2% to the drone total weight, a percent that can be considered almost negligible.

**Figure 30:**
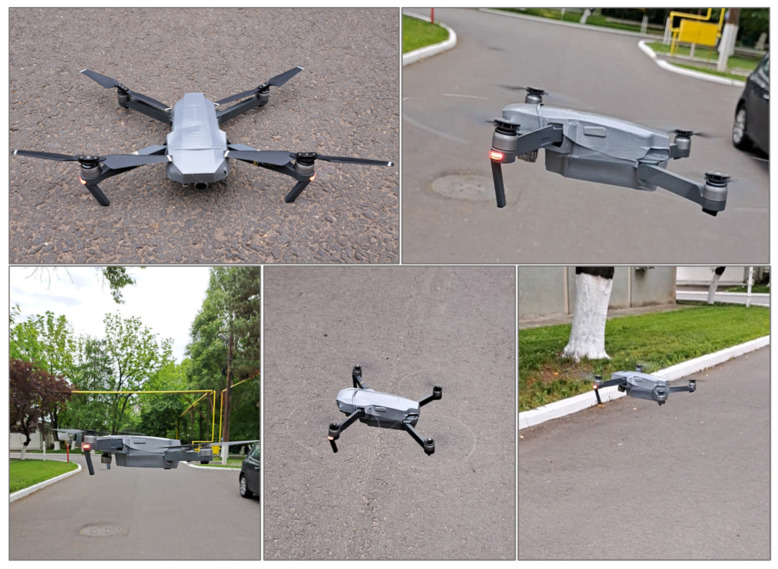
Images captured during the flight test of the drone with the mounted cover from the GFF 15% sample.

The two pieces that compose the cover were attached together using adhesive strips, which allow for easily detaching them and replacing them, but ensure adequate adhesion for the cover to remain in place during flying. After securing the two pieces of the cover, preliminary test flights were conducted, to evaluate the behavior in a dynamic environment (real operating conditions). The tests were conducted in weather conditions relevant to the use of the drone, temperature values of approximately 15 °C, humidity of 60–70%, and wind of 15–19 km/h, over a period of 5–10 min (Figure 30).

All samples successfully withstood the flight tests, maintaining their fixed position on the drone’s body during the entire flight test, without being displaced either by the air currents generated by the rapid rotation of the propellers, nor by those generated by the change in the route or atmospheric conditions (wind).

## 4. Conclusions

This study presents the development, characterization, and testing of thermoplastic composites reinforced with as-received and SiO_2_-surface-modified aramid fibers, and their thermoforming over 3D-printed molds to produce a two-part cover for a small-size recreational drone. This study was composed of three main steps, which developed gradually, validating the results step-by-step.

Within the first experimental step, the FTIR, SEM, and EDS analysis of the surface of the fibers confirmed the successful modification of the aramid fiber by the sol–gel method using TEOS as a precursor, without attesting any damage or defects generated on the surface, unreacted precursor presence, or agglomerated SiO_2_ particles.

Within the second experimental step, composites based on the 50/50 PP/PE-g-MA matrix, 1% Al-based pigment, and 10 and 15 wt% as-received or modified aramid fibers were manufactured by melt-compounding and thermal pressure. The morphostructure analyses performed by SEM and FTIR microscopies indicate a solid incorporation of the SiO_2_-modified fibers into the thermoplastic blend, attributed to the improvement in the interphase through the activation of the fiber surface and establishing interaction between the maleic anhydride in the polymer and the SiO_2_ deposited on the fiber surface. This strong interaction between the phases in the composite leads to a compact and uniform morphology, supporting the mechanical load transfer within the composite, consequently decreasing defects and crack formation and propagation, compared to the control sample and the as-received fiber-based composites. These features lead to improved tensile, flexural, and impact properties, and surface energy values. Thermal testing showed that aramid fibers do not induce negative effects in the operation conditions’ temperature range; moreover, the presence of SiO_2_ in the modified fiber-based composites acts as a stabilizing agent that takes over part of heating energy from the rest of the sample, indicating an improved thermal behavior. Although water contact angle measurements tended to decrease for modified fiber-reinforced samples, this is due to the higher adhesion between the phases and the higher surface energy, all materials exhibited hydrophobic character, with the water contact angle higher than 100°. Mechanical properties’ measurements indicated an average increase in tensile strength of composites with modified fibers compared to control samples by approximately 20%, and a 36–52% increase in Young’s modulus, with higher values for room temperature conditions. In terms of flexural properties, modified fiber-based composites showed an average increase by 26–33% for strength and 30–47% for the modulus, with higher values for room temperature conditions. Impact resistance showed an increase by 20–40% in the fiber-reinforced composites compared to the control sample.

The third step was the implementation for the target application, consisting of producing a cover or a skin for the body of a small commercial UAV from the materials developed and tested. A two-part mold, designed in Catia software, to be used as thermoforming support was built from a nylon-based material via 3D printing and standard milled polyurethane foam molds were built for comparison. The developed materials were successfully vacuum-thermoformed over the produced molds, but the 3D-printed ones ensured easier demolding, and the obtaining of draglines’ pattern on the wider surface imprinted a supplementary rigidity by design to the cover. All covers successfully withstood the flight test, performed in a standard operating environment of the drone (outside: 15 °C temperature, 60–70% humidity, 15–19 km/h wind), without detaching or displacing by air currents (natural or generated by the vehicle’s flight). Moreover, after the flight test, each cover was easily and rapidly detached from the drone body and replaced with another one.

The developed materials proved their excellent ability and versatility as protective covers of complex shape parts. Their promising properties together with the excellent thermoforming capacity support these materials as potential solutions to build customizable products with design versatility, not only for aerial vehicles’ field, but also for an unlimited number of applications, including other transport industries such as automotive and public transport vehicles, and are also viable for other different fields that would require similar properties and processing routes. The low-cost, rapid, and facile replacement or even reprocessing of damaged thermoformed parts, allowed for by the recyclable nature of the involved materials, together with the versatility offered by the tailorable design ensured by 3D-printed molds, contributes to the attractiveness of the materials and processes approached within this study.

## Figures and Tables

**Table 1:** Composition of investigated materials, wt%.

Sample Code/Raw Materials	GO1%	GFN 10%	GFN 15%	GFF 10%	GFF 15%
PP K948	50	50	50	50	50
PE-g-MA (Adamer)	50	50	50	50	50
Opaque gray pigment—Mastersafe MP-10-20B	1	1	1	1	1
Unmodified aramid fibers	-	10	15		
Aramid fibers modified with SiO_2_ (TEOS precursor)	-	-	-	10	15
Polyethylene-based wax (relative to the fiber amount)	-	5	5	5	5

**Table 2:** The characteristics of Onyx material, as per manufacturer measurements reported in the datasheet.

Composite Base Test (ASTM)	Onyx Characteristics
Tensile Modulus (GPa) D638	2.4
Tensile Stress at Yield (MPa) D638	40
Tensile Stress at Break (MPa) D638	37
Tensile Strain at Break (%) D638	25
Flexural Strength (MPa) D790	71
Flexural Modulus (GPa) D790	3
Heat Deflection Temp (°C) D648 B	145
Izod Impact Strength-Notched (J/m) D256-10 A	330
Density (g/cm^3^)	1.2

**Table 3:** The parameters used for 3D printing the two mold pieces that compose the cover mold.

Parameter	Upper Mold	Lower Mold
Print time, h: min	8:37	19:23
Layer height, mm	0.25	0.2
Infill pattern	Hexagonal	Hexagonal
Infill density, %	27	18
Roof and floor no. of layers (height, mm)	3 (0.75)	7 (0.80)
Wall no. of layers (height, mm)	2 (0.80)	2 (0.80)
Total no. of layers	163	274
Mass, g	141.86	161.47
Plastic volume, cm^3^	131.56	147.75

**Table 4:** Average tensile properties measured at room temperature and +39 °C.

Sample Codification	Tensile Strength, MPa	Tensile Modulus, MPa	Tensile Strain, %
Test condition—Room temperature			
GO1%	7.3 ± 0.34	625.45 ± 109.26	18.36 ± 3.26
GFN 10%	8.46 ± 0.19	832.08 ± 49.78	12.74 ± 0.21
GFN 15%	8.61 ± 0.36	1037.31 ± 32.95	7.5 ± 0.13
GFF 10%	8.43 ± 0.16	788.28 ± 20.72	19.87 ± 1.58
GFF 15%	9.09 ± 0.12	917.33 ± 38.02	7.97 ± 0.39
Test condition—+39 °C			
GO1%	7.14 ± 0.13	457.97 ± 65.54	52.54 ± 1.70
GFN 10%	7.66 ± 0.20	579 ± 19.98	24.64 ± 3.00
GFN 15%	8.55 ± 0.20	1020.11 ± 195.06	13.94 ± 0.20
GFF 10%	8.39 ± 0.42	641.73 ± 9.72	11.63 ± 2.34
GFF 15%	9.12 ± 0.05	753.9 ± 10.83	15.55 ± 0.18

**Table 5:** Average flexural properties measured at room temperature and +39 °C.

Sample Codification	Flexural Strength, MPa	Flexural Modulus, MPa	Flexural Strain, %
Test condition—Room temperature			
GO1%	10.62 ± 0.17	601.45 ± 14.48	3.12 ± 0.23
GFN 10%	13.8 ± 0.20	838.76 ± 91.58	2.74 ± 0.05
GFN 15%	13.43 ± 0.23	974.29 ± 34.78	2.93 ± 0.01
GFF 10%	13.81 ± 0.13	847.97 ± 33.46	2.74 ± 0.00
GFF 15%	14.54 ± 0.18	918.37 ± 5.91	2.64 ± 0.23
Test condition—+39 °C			
GO1%	7.13 ± 0.14	460.86 ± 1.04	2.9 ± 0.11
GFN 10%	7.75 ± 0.08	572.17 ± 1.35	2.74 ± 0.13
GFN 15%	8.78 ± 0.03	621 ± 73.98	3.13 ± 0.08
GFF 10%	8.29 ± 0.01	587.99 ± 25.01	2.89 ± 0.13
GFF 15%	9.62 ± 0.17	614.74 ± 50.85	3.00 ± 0.09

**Table 6:** Principal numerical data from thermal analysis for all five composite samples.

Sample	T5% (°C)	T10% (°C)	T50% (°C)	Mass Loss % RT-250 °C	Melting Peak (°C)	Decomposition Onset (°C)
GO1%	326.9	353.9	400.3	0.38	154.8	165.9
GFN 10%	315.7	360.1	410.8	0.88	155.7	165.1
GFN 15%	304.4	341.9	406.5	1.09	154.7	164.7
GFF 10%	322.3	351.2	415.3	0.80	155.5	165.4
GFF 15%	325.0	354.4	414.7	0.77	155.7	165.7

## Data Availability

The original contributions presented in the study are included in the article, further inquiries can be directed to the corresponding author.
